# Efficacy of SGLT2 Inhibitors, GLP-1 Receptor Agonists, DPP-4 Inhibitors, and Sulfonylureas on Moderate-to-Severe COPD Exacerbations Among Patients with Type 2 Diabetes: A Systematic Review and Network Meta-Analysis

**DOI:** 10.3390/ph18091337

**Published:** 2025-09-05

**Authors:** Edoardo Pirera, Domenico Di Raimondo, Lucio D’Anna, Antonino Tuttolomondo

**Affiliations:** 1 Internal Medicine and Stroke Care Ward, Department of Promoting Health, Maternal-Infant, Excellence and Internal and Specialized Medicine (Promise) G. D’Alessandro, University of Palermo, Piazza delle Cliniche 2, 90127 Palermo, Italy; domenico.diraimondo@unipa.it (D.D.R.); bruno.tuttolomondo@unipa.it (A.T.); 2 Department of Stroke and Neuroscience, Charing Cross Hospital, Imperial College London NHS Healthcare Trust, Fulham Palace Rd, London W6 8RF, UK; l.danna@imperial.ac.uk

**Keywords:** COPD, chronic obstructive pulmonary disease, cardiopulmonary risk, type 2 diabetes, GLP-1 receptor agonists, DPP4 inhibitors, SGLT2 inhibitors, sulfonylureas

## Abstract

**Background/Objectives**: Chronic obstructive pulmonary disease (COPD) and type 2 diabetes mellitus (T2DM) frequently coexist, contributing to worse clinical outcomes and increased risk of exacerbations. While newer glucose-lowering agents have demonstrated cardiovascular and renal benefits, their comparative efficacy on COPD exacerbations remain uncertain. **Methods**: We systematically searched PubMed, Embase, Web of Science, Cochrane Library, and ClinicalTrials.gov from inception to June 2025. We included randomised controlled trials (RCTs) and observational studies enrolling adults with COPD and T2DM that reported the risk of COPD exacerbations following initiation of SGLT2is, GLP-1RAs, DPP-4is, or sulfonylureas, with an active comparator group. The primary outcome was a composite of moderate-to-severe COPD exacerbations. Secondary outcomes included the individual components separately. A Bayesian random-effects network meta-analysis was performed to estimate risk ratio (RR) with 95% credible intervals (95% CIs). **Results**: Nine observational studies were ultimately included. No RCTs were retrieved. Compared to sulfonylureas, initiation of SGLT2is (RR 0.64, 0.59–0.69), GLP-1RAs (0.66, 0.60–0.71), and DPP-4is (0.79, 0.74–0.86) was associated with reduced risk of moderate-to-severe exacerbations. Moreover, SGLT2is (0.80, 0.75–0.86) and GLP-1RAs (0.83, 0.77–0.88) were more favourable compared to DPP4is. Consistent results were found for secondary outcomes. Sensitivity analyses confirmed the robustness of the findings for the primary outcome. Robustness was not consistently observed across all treatment comparisons for secondary outcomes. **Conclusions**: Among patients with COPD and T2DM, newer glucose-lowering agents, particularly SGLT2is and GLP-1RAs, were associated with significantly lower risk of moderate-to-severe exacerbations. These findings support the potential respiratory benefits of these agents and warrant confirmation through RCTs.

## 1. Introduction

Chronic obstructive pulmonary disease (COPD) is a chronic respiratory condition characterised by progressive airflow limitation and persistent respiratory symptoms. Being the third leading cause of morbidity and mortality worldwide, COPD is a major public health challenge and places a significant burden on healthcare systems [1,2]. It is estimated that COPD is responsible for approximately 3 million deaths worldwide each year [3]. Projections further suggest that the rising prevalence of smoking in low- and middle-income countries, together with the aging population in high-income nations, could drive the number of deaths attributable to COPD and related conditions to exceed 5.4 million annually by 2060 [4,5].

COPD frequently occurs in the context of multimorbidity, with cardiovascular diseases (CVDs) being the leading contributor, creating a syndemic scenario which exacerbates clinical outcomes, complicates treatment management, and further increases the use of healthcare resources. Among the CVDs associated with COPD, type 2 diabetes mellitus (T2DM) holds particular clinical relevance. In clinical practice, the co-occurrence of T2DM and COPD is frequently observed: approximately 10% of patients with T2DM also suffer from COPD, whereas individuals with COPD exhibit a 1.33-fold increased risk of developing T2DM compared to those without COPD [6,7]. Furthermore, among patients with COPD, T2DM contributes independently to worse pulmonary outcomes, including accelerated lung function decline and increased frequency and severity of exacerbations [8,9,10,11].

Recent clinical studies showed that newer glucose-lowering medications, namely sodium-glucose cotransporter-2 inhibitors (SGLT2is), glucagon-like peptide-1 receptor agonists (GLP-1RAs), and dipeptidyl peptidase-4 inhibitors (DPP-4is), offer additional therapeutic benefits beyond glycemic control, particularly in reducing cardiovascular and renal events [12]. Emerging evidence further suggests that these agents may beneficially impact respiratory function and COPD outcomes through mechanisms such as reducing systemic carbon dioxide levels, modulation of airway and systemic inflammation, reduction in bronchial hyperresponsiveness, and systemic metabolic improvements [13,14,15,16]. However, evidence comparing the impact of newer glucose-lowering therapies on respiratory outcomes remains limited. Given the increasing recognition of COPD as part of a cardiopulmonary syndemic, a comprehensive assessment of their relative efficacy in exacerbating COPD risk is timely and clinically relevant.

Given these evidence gaps, we conducted a systematic review and network meta-analysis (NMA) of observational studies aimed to explore the comparative efficacy of SGLT2is, GLP-1RAs, DPP-4is, and sulfonylureas in preventing moderate-to-severe COPD exacerbations among patients with T2DM.

## 2. Methods

### 2.1. Search Strategy, Selection Criteria, and Data Extraction

We systematically searched for peer-reviewed reports with no language restriction in Embase, PubMed, Cochrane databases, WebOfScience, and ClinicalTrials.gov from inception to 16 April 2025 and updated searches were re-run to 10 June 2025. The search term strategy included a combination of synonyms of “COPD” and each T2DM medication. The search strategy for each database is fully reported in Supplementary Table S1. This systematic review and meta-analysis was registered on the International Prospective Register of Systematic Reviews (PROSPERO, CRD420251016904) and reported in accordance with the Preferred Reporting Items for Systematic Review and Meta-Analysis (PRISMA) statement guidelines and with the Meta-analyses Of Observational Studies in Epidemiology (MOOSE) statement [17]. PRISMA-NMA and MOOSE checklists are available in Supplementary Table S2 and Table S3, respectively [18]. For the purpose of this meta-analysis, we included studies that met all of the following eligibility criteria: (1) randomised controlled trials (RCTs) and observational studies; (2) including adult patients with a COPD diagnosis and T2DM; (3) studies that assessed the association between the initiation of glucose-lowering medications—including SGLT-2is, GLP-1RAs, DPP-4is, and sulfonylureas—and the outcome of interest; (4) presence of an active comparator. Editorials, letters to editor, conference abstracts, or any studies that have not been peer-reviewed were excluded from the qualitative and quantitative analysis. Potential eligibility of the records identified from the literature search were independently assessed by two authors (EP and AT) according to the eligibility criteria and with a two-step inspection (title/abstract and full-text review). Disagreement was resolved by consensus between a third author (LDA). Data extraction was independently performed by two authors (EP and DDR) and reported into an electronic spreadsheet. Disagreements in data extraction were resolved through discussion between the two authors. In cases of persistent disagreement, a third reviewer provided a final opinion to reach consensus (AT).

### 2.2. Endpoint Definition

The primary endpoint was a composite of moderate or severe COPD exacerbations. Secondary outcomes included the individual components of the composite endpoint. Specifically, moderate exacerbations were defined as an outpatient visit for COPD associated with a prescription for systemic corticosteroids with or without antibiotics, and not requiring hospitalization. Severe exacerbations were defined as hospital admissions or emergency department visits [1].

### 2.3. Risk of Bias Assessment

The risk of bias of included studies was assessed using the “Risk Of Bias In Non-randomized Studies of Interventions” (ROBINS-I) tool [19]. This tool evaluates risk of bias across seven domains: bias due to confounding, selection of participants, classification of interventions, deviations from intended interventions, missing data, measurement of outcomes, and selection of the reported result. Each domain was judged as having low, moderate, serious, or critical risk of bias, or no information. Two reviewers (EP and DDR) independently assessed each study, with discrepancies resolved through discussion with a third reviewer.

### 2.4. Statistical Analysis

A Bayesian NMA model was fitted in order to simultaneously compare the initiation of regimens of interests. From eligible studies, we extracted the sample size and total number of events for each of the predefined outcomes in each treatment group. To account for effect heterogeneity across trials, we performed the NMA under a random-effects model. Network geometry was visually represented using network plots, in which nodes represent treatments and edges represent available direct comparisons. Risk ratios (RRs) for the effects of both regimens and their corresponding 95% credible intervals (95% CIs) were estimated using Markov chain Monte Carlo method. All analyses were performed using the gemtc package in R, version 4.4.1 (The R Foundation). The NMA was conducted using the package’s default configuration, which applies noninformative prior distributions and runs four independent Markov chains, each generating 50,000 posterior samples following a 20,000-iteration burn-in phase. Convergence of the Markov chain Monte Carlo algorithms for all model parameters was assessed using trace plots and the Gelman–Rubin diagnostic. We employed rank probabilities to assess the ranking of each intervention, and calculated the surface under the cumulative ranking curve (SUCRA) values to provide a measure of treatment efficacy, where higher SUCRA values indicate greater probability that a treatment ranks among the most effective options [20]. SUCRA values range from 0 (treatment is certainly the least effective) to 1 (treatment is certainly the most effective). We conducted a qualitative assessment of baseline participant characteristics—such as cardiovascular diseases, COPD therapy, COPD severity, and exacerbation history—to verify that the transitivity assumption was met across treatment comparisons. To assess the robustness of our results, we performed several sensitivity analyses: (1) a frequentist approach; (2) a node-splitting approach; (3) a comparison of Bayesian model fit with and without the assumption of evidence inconsistency [21,22]; (4) a comparison of Bayesian model fit using different prior distributions [23]; (5) NMA using adjusted effect estimates reported in the included studies, instead of sample size and total number of events; (6) NMA including only studies judged at “low risk” of bias according to ROBINS-I. Heterogeneity was assessed with I^2^ statistic and the standard deviation of the log risk ratio scale (τ). According to the Cochrane Collaboration, we considered different degrees of heterogeneity as follows: an I^2^ value of 0–40% indicates low heterogeneity; 30–60% moderate heterogeneity; 50–90% substantial heterogeneity; 75–100% considerable heterogeneity [24]. The τ was employed to assess the consistency and robustness of the models, including the comparison of Bayesian model fit with and without the assumption of evidence inconsistency, as well as across models fitted with different prior distributions.

## 3. Results

### 3.1. Literature Search and Study Characteristics

**Figure 1:**
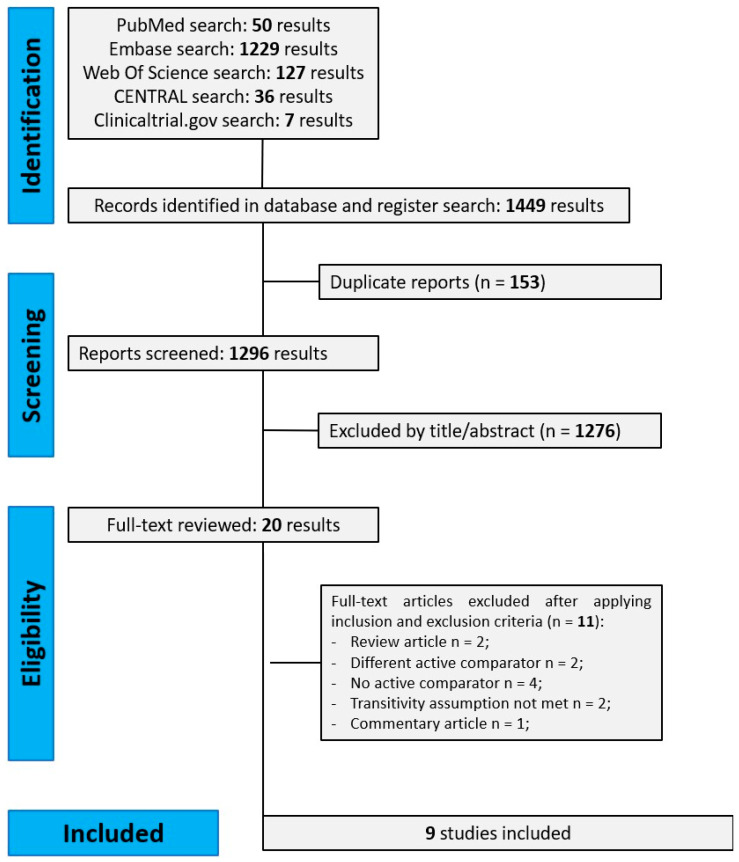
Flow-chart of study selection and reasons for exclusion according to PRISMA Guidelines.

Our literature search yielded 1450 results. After removing duplicates (n = 153) and ineligible studies based on title/abstract inspection (n = 1276), we fully reviewed 20 studies. We finally included nine observational studies in the systematic review and quantitative analysis [25,26,27,28,29,30,31,32,33]. No RCTs were retrieved. The study selection process is shown in Figure 1 and reasons for exclusion are briefly reported in Supplementary Table S4. Follow-up duration ranged from 6 months to 2.5 years. Characteristics of included studies are shown in Table 1 and baseline characteristics of participants, including CVD history, glucose-lowering regimens, COPD therapy, severity, and exacerbation history are reported in Supplementary Table S5. Based on a qualitative assessment of these baseline characteristics across treatment comparisons, the transitivity assumption was reasonably satisfied. Risk of bias judgment is reported in Supplementary Table S6.

### 3.2. Structure of NMA

**Figure 2:**
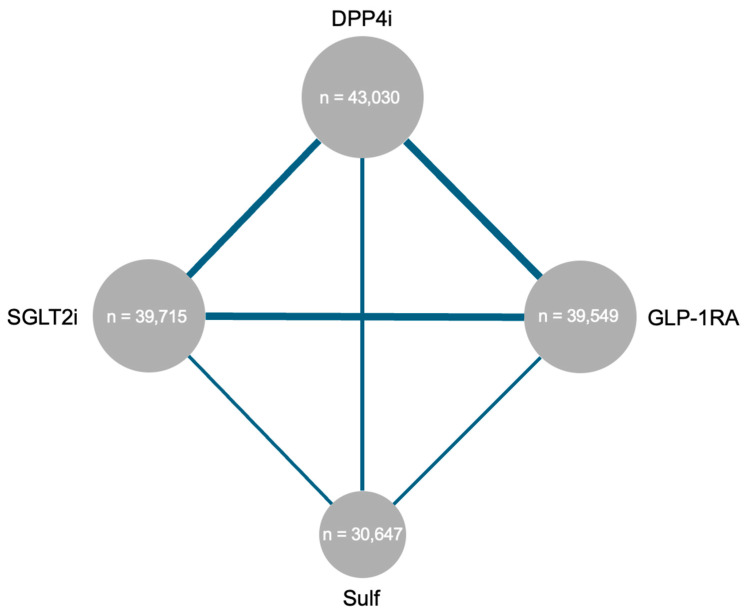
Geometry of network meta-analysis for the primary outcome. The network plot shows the glucose-lowering agents evaluated in the network meta-analysis. Each node represents a specific glucose-lowering class, with node size proportional to the number of subjects receiving that treatment across the included studies. The connecting lines between nodes indicate direct comparisons from studies, with line thickness proportional to the number of subjects involved in those direct comparisons. Abbreviations: DPP4i: dipeptidyl peptidase-4 inhibitor; GLP-1RA: glucagon-like peptide-1 receptor agonist; SGLT2i: sodium-glucose cotransporter-2 inhibitor; Sulf: sulfonylurea.

Figure 2 shows the network geometry for the primary outcome and Supplementary Figures S1 and S2 show the networks for the secondary outcomes. For the primary outcome, three direct comparisons were available between DPP4is and both GLP-1RAs and SGLT2is, with two comparisons for the other treatment regimens. For moderate exacerbations, three direct comparisons were available for DPP4is vs. SGLT2is and two direct comparisons for the remaining treatment regimens. Regarding severe exacerbations, five direct comparisons were available between DPP4is and SGLT2is, three comparisons each for DPP4is versus GLP-1Ras, GLP-1RAs versus SGLT2is, SGLT2is, and sulfonylureas, and two comparisons for the remaining treatment regimens.

### 3.3. NMA Results for the Primary and Secondary Outcomes

**Figure 3:**
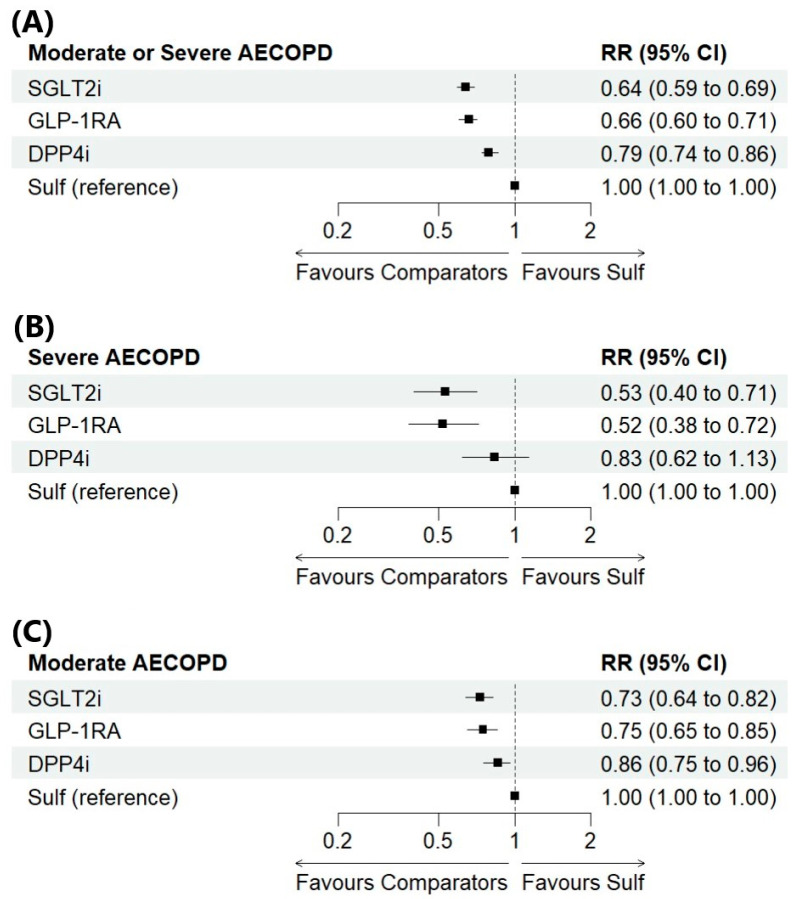
Forest plots for “moderate or severe” (**A**), “severe” (**B**) and “moderate” (**C**) COPD exacerbation. Data are reported as risk ratios and 95% credible interval. Abbreviations: AECOPD: acute exacerbations of chronic obstructive pulmonary disease; DPP4i: dipeptidyl peptidase-4 inhibitor; GLP-1RA: glucagon-like peptide-1 receptor agonist; RR: Risk ratio; SGLT2i: sodium-glucose cotransporter-2 inhibitor; Sulf: sulfonylurea; 95% CI: 95% credible interval.

Five studies with a total of 152.941 individuals comparing DPP4is, GLP-1RAs, SGLT2is, and sulfonylureas were included in the network meta-analysis for the primary outcome (Figure 3A). Compared with sulfonylureas, new users of SGLT2is (RR: 0.64, 95% CI 0.59–0.69), GLP-1RAs (RR: 0.66, 95% CI 0.60–0.71), and DPP4is (RR: 0.79, 95% CI 0.74–0.86) had a significantly lower risk of moderate or severe COPD exacerbations with low heterogeneity (I^2^: 8%). Furthermore, both SGLT2is (RR, 0.80; 95% CI, 0.75–0.86) and GLP-1RAs (RR, 0.83; 95% CI, 0.77–0.88) were associated with a lower risk compared to DPP4is. No significant difference was observed between GLP-1RAs and SGLT2is.

Eight studies involving 184.574 individuals and four studies involving 149.439 individuals were included in the NMA for the secondary outcomes of severe (I^2^: 4%) and moderate (I^2^: 0%) COPD exacerbations, respectively (Figure 3B and Figure 3C). Compared with sulfonylureas, treatment initiation of GLP-1Ras and SGLT2is were associated with a significantly lower risk of severe and moderate exacerbations of COPD. New users of DPP4is also had a lower risk of moderate COPD exacerbations compared to sulfonylureas, although no significant difference was observed for severe exacerbations of COPD. Additionally, SGLT2is and GLP-1RAs were confirmed to be favourable compared to DPP4is for both the secondary outcomes. No significant differences were found between new users of GLP-1RAs and SGLT2is for both the secondary outcomes.

Pairwise comparisons among glucose-lowering treatments for the primary and secondary outcomes are presented in Supplementary Figure S3. Diagnostic statistics of the main analysis are reported in Supplementary Figure S4.

### 3.4. Ranking of Treatment Strategies

Table 2 shows SUCRA values for the outcomes analysed. For moderate or severe exacerbations, SGLT2is ranked highest (SUCRA: 0.938), followed by GLP-1RAs (0.729), DPP4is (0.333), and sulfonylureas (0.000). For severe exacerbations, GLP-1RAs achieved the highest SUCRA value (0.847), followed by SGLT2is (0.818) and DPP4is (0.299), with sulfonylureas (0.035) ranking lowest. Similarly, for moderate exacerbations, SGLT2is ranked first (0.892), followed by GLP-1RAs (0.768), DPP4is (0.337), and sulfonylureas (0.002). Overall, SGLT2is and GLP-1RAs consistently ranked as the most effective therapies, with DPP4is and sulfonylureas demonstrating lower relative effectiveness across all outcomes.

### 3.5. Results of Sensitivity Analyses

To assess the robustness of our NMA model, we performed several sensitivity analyses (Supplementary S1). First, node-splitting analysis showed neglectable evidence of inconsistency for all fitted models, except for the comparison of sulfonylureas and DPP4is for severe COPD exacerbation. Second, comparisons of Bayesian model fit with and without the assumption of evidence inconsistency and using different prior distributions showed similar effect estimates. Third, both the frequentist analysis and the NMA using reported effect sizes yielded consistent results with our main analysis. Notably, the comparison between SGLT2is and GLP-1RAs reached statistical significance in these analyses but not in the main analysis. For severe COPD exacerbations, results remained robust, while for moderate COPD exacerbations results were not consistent in the DPP4is versus SGLT2is and versus sulfonylureas comparisons. Finally, the NMA including only “low risk” studies confirmed the robustness of the results for the primary outcome, while for severe exacerbations, there was a loss of significance between DPP4i and GLP-1RA. A loss of significance was observed for moderate exacerbations in most comparisons.

## 4. Discussion

In this Bayesian NMA including COPD patients with T2DM, initiation of SGLT2is, GLP-1RAs, and DPP4is was associated with a 36%, 34%, and 21% lower risk of moderate or severe COPD exacerbations compared to sulfonylureas, respectively. Additionally, compared to initiation of DPP4is, SGLT2is and GLP-1RAs appear to decrease the likelihood of the primary outcome by 20% and 17%, respectively. These results remained consistent across the sensitivity analyses, except for the head-to-head comparison between SGLT2is and GLP-1RAs that showed a 7% lower risk of moderate or severe COPD exacerbations.

For severe COPD exacerbations, SGLT2is and GLP-1RAs showed a 47% and 48% lower risk, respectively, compared to sulfonylureas. No differences were found between DPP4is and sulfonylureas and between SGLT2is and GLP-1RAs. This finding remained robust across the sensitivity analyses. Compared to initiation of DPP4is, SGLT2is and GLP-1RAs were associated with a 36% and 37% reduction in the risk of severe exacerbation, remaining consistent throughout the sensitivity analyses.

Our results also suggest that initiation of SGLT2is, GLP-1Ras, and DPP4is was associated with a 27%, 25%, and 14% lower risk of moderate exacerbations, compared to sulfonylureas, respectively. Sensitivity analyses confirmed these findings except for the head-to-head comparison between DPP4is and sulfonylureas. Additionally, our findings suggest that SGLT2is and GLP-1RAs may be associated with a lower risk of moderate exacerbations of COPD compared to DPP-4is. Sensitivity analysis including only “low risk” studies showed a loss of significance in most comparisons.

The current understanding of how SGLT2is, GLP-1Ras, and DPP4is reduce the risk of COPD exacerbations is still limited and remains an area of active research. GLP-1RAs appear to exert modulatory effects on airway smooth muscle responsiveness via suppression of specific inflammatory cytokine pathways, notably those involving IL-13 and IL-33 [35,36,37] and a bronchorelaxant activity mediated by activation of the cAMP-PKA cascade, the same intracellular pathway activated by β2-adrenergic receptor agonists [38]. Additionally, administration of GLP-1RAs to patients with T2DM has been shown to improve forced expiratory volume in 1 s (FEV_1_) and increase forced vital capacity (FVC) above the minimal clinically important difference (MCID) of 100 mL [39], whereas in obese COPD patients, treatment resulted in significant weight loss, increased FVC and carbon monoxide diffusing capacity, improved CAT score, but no significant changes in FEV_1_, FEV_1_/FVC, or 6-min walk distance [40]. Similarly to GLP-1RAs, DPP4is have also been reported to reduce bronchial hyperresponsiveness by increasing endogenous GLP-1 by directly blocking DPP-4, a ubiquitous enzyme that selectively degrades GLP-1 and is upregulated in lung-infiltrating inflammatory cells that perpetuate inflammation in patients with COPD [16]. Our NMA showed that the initiation of GLP-1RAs in patients with T2DM was associated with a significantly lower risk of COPD exacerbations compared to DPP4is, aligning with previous studies where GLP-1RAs exhibited greater efficacy in glucose control, and in reducing cardiovascular and renal outcomes [12]. Despite targeting a similar pathway, GLP-1RAs have been shown to increase GLP-1 hormone levels 10-fold, potentially explaining their efficacy compared to DPP4is [41].

While the effects of SGLT2Is on the cardiac and renal systems have been well studied, their role in the respiratory system remains largely unknown. By decreasing serum glucose levels and promoting glucosuria, SGLT2is reduce endogenous carbon dioxide production, which is beneficial for COPD patients [42]. Furthermore, SGLT2is increased urinary excretion of both glucose and sodium, resulting in osmotic diuresis and natriuresis: this mechanism can help manage fluid balance and reduce cardiac workload, which is particularly beneficial in patients with COPD and comorbid heart failure.

Despite the inherent limitations of an analysis based on observational data, the clinical implications of our NMA are of major importance, especially as COPD is increasingly recognised not merely as a pulmonary condition but rather as the respiratory manifestation of systemic chronic inflammation, reflecting a syndemic scenario characterised by a close interaction with cardiovascular comorbidities [43,44]. Current pharmacological management of COPD relies predominantly on inhaled bronchodilators, which are effective in improving symptoms and reducing exacerbations. However, these treatments have not demonstrated efficacy in reducing cardiovascular outcomes. Indeed, cardiovascular events represent the leading cause of mortality among COPD patients and their incidence is markedly increased following acute exacerbations with a well-defined temporal trajectory [45]. This highlights a therapeutic gap in addressing the cardiopulmonary continuum that characterises this population, which our NMA is attempting to fill. Preventing exacerbations is critical in COPD management, as even a single exacerbation may have a negative impact on the patient’s outcome. Current COPD recommendations recognise the role of T2DM as a relevant comorbidity in COPD, but do not appropriately address treatment implications [1]. In this context, the significant reduction in moderate-to-severe COPD exacerbations observed with initiation of SGLT2is and GLP-1RA is of particular therapeutic interest. By reducing exacerbation frequency and severity, newer glucose-lowering drugs might simultaneously confer cardiopulmonary benefits, allowing a holistic approach to COPD, which has been advocated by several authors [46].

Our findings suggest that the use of SGLT2is and GLP-1RAs could be a relevant component of a holistic approach to the treatment of patients with coexisting COPD and T2DM. While a clear mechanistic rationale can explain the superior efficacy of GLP-1RAs over DPP-4i, the comparative effectiveness of GLP-1RAs and SGLT2i is still uncertain. Although our sensitivity analysis suggests that initiation of SGLT2i leads to a significantly lower incidence of COPD exacerbation compared to GLP-1RAs, this finding warrants cautious interpretation given the multiple methodological and clinical limitations of the available evidence. There are various potential explanations of these results: GLP-1RAs exert anti-inflammatory and metabolic effects and are associated with substantial body weight reduction, particularly at higher doses. By contrast, SGLT2is typically induce a more modest weight loss but are consistently associated with improvements in cardiorenal outcomes [47]. Importantly, recent meta-analyses have shown that the weight-lowering effect of GLP-1RAs is less pronounced in patients with T2DM than in those without T2DM, whereas the weight-lowering effect of SGLT2is seems relatively consistent across individuals with or without T2DM [48,49,50]. Since all patients included in our NMA had T2DM, the expected advantage of GLP-1RAs on weight reduction may have been attenuated in this setting. Furthermore, SGLT2is are known to reduce the incidence of heart failure exacerbations, which are often misclassified as COPD exacerbations in routine clinical practice. These factors may at least partly explain why our sensitivity analyses suggested a benefit with SGLT2is over GLP-1RAs, although the finding was not robust in the main NMA and should therefore be interpreted with caution. Future RCTs specifically designed to compare the respiratory efficacy of these glucose-lowering agents are therefore essential to inform clinical decision-making. Further research is warranted to determine whether these therapies yield sustained improvements in pulmonary function and to investigate potential heterogeneity of treatment effects across clinically relevant subgroups—particularly according to COPD severity, history of exacerbations, or coexisting CVD. Such evidence could meaningfully inform therapeutic decision-making and improve the clinical management of patients with coexisting COPD and T2DM.

Our study has several limitations that warrant acknowledgment when interpreting the results. First, the primary limitation is its exclusive reliance on observational studies, which restricts the strength of causal inferences. This was a necessary approach, as our comprehensive systematic search did not identify any RCTs that met our eligibility criteria. Therefore, our NMA represents the best summary of the currently available evidence to date to address this clinical issue. To mitigate the inherent risks of selection and confounding bias associated with observational data, we implemented a rigorous methodological framework. Our protocol mandated the inclusion of studies with a “new-user, active-comparator” design. This design is a well-established strategy to reduce common biases by creating a scenario that more closely emulates the randomization in a clinical trial. Furthermore, most of the included studies utilised propensity score matching or weighting to balance covariates between treatment groups, which further strengthens the validity of the comparisons. While these approaches strengthen validity, residual confounding by unmeasured or incompletely captured factors (e.g., COPD severity, smoking status, adherence to therapy, socioeconomic status) cannot be excluded. Thus, unmeasured confounding remains an important source of uncertainty in the interpretation of our results. Second, clinical and methodological heterogeneity among the included studies may have influenced the results. Variability was observed in baseline patient characteristics (e.g., prevalence of CVD, COPD therapy, and exacerbation history at baseline), follow-up duration, and definitions of moderate and severe exacerbations. Although we employed a random-effects model and performed multiple sensitivity analyses that confirmed the robustness of our main findings, the possibility that underlying heterogeneity contributed to between-study variability cannot be ruled out. Moreover, the limited number of studies contributing to some of the treatment comparisons limited the possibility of formally exploring heterogeneity through subgroup analysis or meta-regression. Third, several methodological constraints should be considered. The geometry of the treatment network was relatively sparse, with few direct comparisons, which reduces the precision of indirect comparisons. The absence of individual patient-level data prevented formal assessment of transitivity and precluded subgroup analyses according to clinically relevant variables (e.g., COPD severity, BMI, baseline HbA1c, heart failure). Moreover, although most studies used validated algorithms for patient and outcome identification, some degree of misclassification remains possible. Finally, due to the limited number of included studies (<10), we could not perform meta-regression, Egger’s test, or funnel plots to assess source of heterogeneity or publication bias, respectively, as such analyses are not considered reliable with their small evidence bases. This decision was made in accordance with the *Cochrane Handbook for Systematic Reviews of Interventions* [24]. These limitations imply that our findings should be interpreted with caution. They provide valuable preliminary insights into the potential respiratory benefits of newer glucose-lowering agents in patients with COPD and T2DM but underscore the urgent need for confirmatory randomised controlled trials specifically designed to address this question.

## 5. Conclusions

In conclusion, the initiation of SGLT2is, GLP-1RAs, and DPP4is was associated with a reduced risk of moderate or severe exacerbations in patients with COPD and T2DM compared to sulfonylureas. Additionally, head-to-head comparisons between novel glucose-lowering agents showed that SGLT2is and GLP-1RAs were associated with a lower risk of moderate or severe COPD exacerbations compared to DPP4is. However, as these findings are derived exclusively from observational data, they should be interpreted with caution, and only limited causal inferences can be drawn. Our results therefore provide valuable preliminary evidence supporting continued investigations of SGLT2is, GLP-1RAs, and DPP4is in this population and highlight the urgent need for confirmatory RCTs, which could ultimately inform treatment algorithms similar to those established for cardiovascular and renal outcomes.

## Figures and Tables

**Table 1:** Characteristics of the studies included in the NMA.

Author and Year	Intervention (n)	Comparator (n)	Age (Mean ± SD)		Male (I:C) ^a^	Body Mass Index ^b^ (I:C) ^a^	Follow-up (Median and IQR)	COPD Severity (FEV_1_% Predicted) ^d^	COPD Exacerbation History
			Intervention	Comparator					
Albogami, 2021 [25]	GLP1-RA (4091)	DPP4i (12,445)	53.6 ± 7.8	53 ± 8	41.1:42.5	N/A	1 year	N/A	Severe AECOPD DPP4i: 2.3; GLP-1RA: 2.3;
Pradhan, 2022 [26]	GLP1-RA (1252)	Sulfonylurea (14,259)	61.4 ± 8.9	61.1 ± 9.2	50:50	<30: 6:6.6	1 year (0.4–2.3)	GLP-1RA <30: 2.6; 30–80: 55.8; >80: 12.5; Missing: 29.2; DPP4i <30: 2.9; 30–80: 58; >80:14.1; Missing: 25; SGLT2i <30: 1.8; 30–80: 57.9; >80: 14.7; Missing: 25.6; Sulfonylurea <30: 3.5; 30–80: 57.4; >80: 11.8; Missing: 27.3;	Severe AECOPD GLP-1RA: 25.8; DPP4i: 26.9; SGLT2i: 21;
						≥30: 91.6:90.0			
	DPP4i (8731)	Sulfonylurea (18,204)	69.3 ± 10.7	68.8 ± 10.7	55.4:55.2	<30: 35.1:34.3	1 year (0.4–2.2)		
						≥30: 63.9:64.7			
	SGLT2i (2956)	Sulfonylurea (10,841)	62.9 ± 9.0	62.7 ± 9.1	58.5:58.0	<30: 20.8:21.7	0.9 year (0.3–2.0)		
						≥30: 78.3:77.4			
Foer, 2023 [27]	DPP4i (260)	GLP1-RA (328)	72.2 ± 11.8 ^c^	67.2 ± 9.6 ^c^	61.1:57.6	29.7:34.9 ^e^	6 months	DPP4i GOLD 1: 12.3; GOLD 2: 53.7; GOLD 3: 28.7; GOLD 4: 5.3; GLP-1RA GOLD 1: 13.7; GOLD 2: 52.6; GOLD 3: 30; GOLD 4: 3.8; SGLT2i GOLD 1: 12.9; GOLD 2: 52.6; GOLD 3: 31.2; GOLD 4: 3.3; Sulfonylurea GOLD 1: 11.8; GOLD 2: 49.9; GOLD 3: 29.8; GOLD 4: 8.5;	Number of exacerbations ≤12 months. n (SD) DPP4i: 1.1 (2.1); GLP-1RA: 1.13 (2.04); SGLT2i: 1.36 (2.32); Sulfonylurea: 1.24 (2.21);
	SGLT2i (353)	GLP1-RA (328)	71.5 ± 10.0 ^c^	67.2 ± 9.6 ^c^	67.1:57.6	30.8:34.9 ^e^			
	Sulfonylurea (701)	GLP1-RA (328)	70.1 ± 9.5 ^c^	67.2 ± 9.6 ^c^	59.3:57.6	30.7:34.9 ^e^			
Au, 2023 [28]	DPP4i (1524)	SGLT2i (381)	62.3 ± 10.8	62.2 ± 10.7	51.0:52.0	N/A	DPP4i: 2.3 (1.0–3.5) SGLT2i: 1.5 (0.5–3.0)	N/A	N/A
See, 2024 [29]	GLP1-RA (1751)	DPP4i (1751)	68.2 ± 8.7	68.3 ± 9.7	52.7:52.4	Overweight: 20.9:20.9 Class I: 26.8:25.6 Class II: 19.6:18.6 Class III–IV: 15.2:15.4	1 year	Mean (SD) GLP-1RA: 61.9 (17.2); DPP4i: 61.7 (19.7);	N/A
Yen, 2024 [30]	GLP1-RA (7506)	SGLT2i (7506)	58.6 ± 9.8	58.7 ± 9.4	42.5:42.5	≥ 30: 9.18:9.29	GLP1-RA: 2.51 years SGLT2i: N/A	N/A	≥2 Moderate AECOPD SGLT2i: 30.4; GLP-1RA: 31.07; ≥2 Severe AECOPD SGLT2i: 1.95; GLP-1RA: 2.03;
Chang, 2025 [31]	SGLT2i (188)	DPP4i (181)	<70 year: 33.5% 70–79 years: 42.5% ≥80 years: 23.9%	<70 year: 34.8% 70–79 years: 41.9% ≥80 years: 23.2%	97.9:96.1	N/A	1 year	N/A	≥2 Moderate AECOPD DPP4i: 6.63; SGLT2i: 1.60; ≥2 severe AECOPD DPP4i: 17.68; SGLT2i: 8.51;
Ray, 2025 [32]	SGLT2i (27,991)	DPP4i (27,991)	70.5 ± 8.6	70.7 ± 8.8	50.8:50.5	≥30: 39.7:39.7	Composite SGLT2i: 145 days (61–335) DPP4i: 147 days (62–336) Severe SGLT2i: 150 days (67–354) DPP4i: 147 days (69–356)	N/A	Moderate/severe AECOPD SGLT2i: 4.4; GLP-1RA: 4.1; DPP4i: 4.4; Severe AECOPD SGLT2i: 2.5; GLP-1RA: 2.4; DPP4i: 2.5; Moderate AECOPD SGLT2i: 8.5; GLP-1RA: 8.0; DPP4i: 8.3;
	GLP1-RA (32,107)	DPP4i (32,107)	70.4 ± 8.5	70.4 ± 8.2	45.1:45.1	≥30: 45.2:45.1	Composite GLP1-RA: 142 days (63–339) DPP4i: 147 days (62–336) Severe GLP1-RA: 147 days (69–356) DPP4i: 164 days (73–377)		
	SGLT2i (36,218)	GLP1-RA (36,218)	69.7 ± 8.7	69.7 ± 8.7	48.1:47.9	≥30: 46.1:46.1	Composite SGLT2i: 141 days (61–316) GLP1-RA: 139 days (65–314) Severe SGLT2i: 147 days (67–329) GLP1-RA: 144 days (70–331)		
Yen, 2025 [33]	DPP4i (452)	SGLT2i (452)	60.4 ± 9.7	60.1 ± 10.1	57.1:57.7	≥30: 4.9:7.1	DPP4i: N/A SGLT2i: 2.61 years	N/A	Moderate AECOPD SGLT2i: 31.2; DPP4i: 30.3; Sulfonylurea: 27.5; Severe AECOPD SGLT2i:37.6; DPP4i: 36.7; Sulfonylurea: 32.7;
	Sulfonylurea (312)	SGLT2i (312)	61.2 ± 10.4	60.7 ± 10.2	50.6:50.6	≥30: 8.3:8.6	Sulfonylurea: N/A SGLT2i: 2.61 years		

Data are reported as percentage (%), unless otherwise stated. Efforts were made to report all available data and where data were unavailable, the most informative metrics were provided. ^a^: Proportion expressed as a percentage in the intervention (I) versus comparator (C); ^b^: Body mass index is weight in kilograms divided by height in meters squared (kg/m^2^) and categorised as overweight (25.0–29.9 kg/m^2^), obesity class I (30.0–34.9 kg/m^2^), class II (35.0–39.9 kg/m^2^), and class III (≥40.0 kg/m^2^); ^c^: Mean was estimated from reported median and interquartile range according to Luo et al. [34]; ^d^: GOLD severity refers to the grade of airflow limitation: (1) mild or GOLD 1: Forced Expiratory Volume in the first second (FEV_1_) ≥ 80 predicted; (2) moderate or GOLD 2: 50% ≤ FEV_1_ ≤ 80% of predicted; (3) severe or GOLD 3: 30% ≤ FEV_1_ ≤ 49% of predicted; (4) very severe or GOLD 4: FEV_1_ ≤ 29% of predicted; ^e^: Foer et al. reported BMI as mean; Abbreviations: AECOPD: acute exacerbation of chronic obstructive pulmonary disease; COPD: Chronic obstructive pulmonary disease; DPP4i: dipeptidyl peptidase-4 inhibitor; GLP1-RA: glucagon-like peptide-1 receptor agonist; IQR: interquartile range; I:C: intervention vs. comparator; n: sample size; SD: standard deviation; SGLT2i: sodium-glucose cotransporter-2 inhibitor.

**Table 2:** SUCRA values for each glucose-lowering treatment and outcome ^a^.

Values	SGLT2i	GLP-1RA	DPP4i	Sulf
Moderate or severe AECOPD	0.938	0.729	0.333	0.000
Severe AECOPD	0.818	0.847	0.299	0.035
Moderate AECOPD	0.892	0.768	0.337	0.002

^a^ SUCRA values are displayed as percentage of area under the cumulative rank probability curve. The higher the SUCRA values, the better the treatment performance for the specific outcome. Abbreviations: AECOPD: acute exacerbation of COPD; DPP4i: dipeptidyl peptidase-4 inhibitor; GLP-1RA: glucagon-like peptide-1 receptor agonist; SGLT2i: sodium-glucose cotransporter-2 inhibitor; SUCRA: Surface Under the Cumulative Ranking; Sulf: sulfonylurea.

## Data Availability

The data supporting this study are available in the original publications of the included studies..

## Supplementary Materials

The following supporting information can be downloaded at https://www.mdpi.com/article/10.3390/ph18091337/s1, Table S1: Search strategy for each database; Table S2: PRISMA-NMA checklist; Table S3: MOOSE checklist for meta-analyses of observational studies; Table S4: Reason for exclusion at full-text screening; Table S5: Baseline characteristics of study participants; Table S6: Risk of bias assessment according to ROBINS-I; Figure S1: Network geometry for “severe COPD exacerbations”; Figure S2: Network geometry for “moderate COPD exacerbations”; Figure S3: Summary estimates of primary and secondary outcomes; Figure S4: Diagnostic statistic of NMA—Trace Plots and Gelman-Rubin Plots; Supplementary S1: Results of sensitivity analyses. References [17,18,19,23,25,26,27,28,29,30,31,32,33,51] are cited in the supplementary materials.

## Abbreviations

The following abbreviations are used in this manuscript:

COPD	Chronic Obstructive Pulmonary Disease
CVD	Cardiovascular Disease
DPP4is	Dipeptidyl Peptidase-4 Inhibitor
FEV_1_	Linear Dichroism
FVC	Forced Vital Capacity
GLP-1RAs	Glucagon-Like Peptide-1 Receptor Agonist
MOOSE	Meta-Analyses Of Observational Studies In Epidemiology
NMA	Network Meta-Analysis
PROSPERO	International Prospective Register Of Systematic Reviews
RCT	Randomised Controlled Trial
ROBINS-I	Risk Of Bias In Non-Randomised Studies Of Interventions
RR	Risk Ratio
SGLT2is	Sodium-Glucose Cotransporter-2 Inhibitors
SUCRA	Surface Under The Cumulative Ranking Curve
T2DM	Type 2 Diabetes Mellitus
95% CI	95% Credible Interval
