# Supplementary material for: Efficacy of SGLT2 Inhibitors, GLP-1 Receptor Agonists, DPP-4 Inhibitors, and Sulfonylureas on Moderate-to-Severe COPD Exacerbations Among Patients with Type 2 Diabetes: A Systematic Review and Network Meta-Analysis
Source: Pharmaceuticals (Basel). 2025 Sep 5;18(9):1337. doi: 10.3390/ph18091337 (PMC12472923; doi:10.3390/ph18091337)
Supplement: Supplementary file 1 [file pharmaceuticals-18-01337-s001.zip › pharmaceuticals-3822803-supplementary.pdf]

Supplementary Materials of "EFFICACY OF SGLT2 INHIBITORS, GLP-1 RECEPTOR AGONISTS, DPP-4 INHIBITORS, AND SULFONYLUREAS ON MODERATE-TO-SEVERE COPD EXACERBATIONS AMONG PATIENTS WITH TYPE 2 DIABETES: A SYSTEMATIC REVIEW AND NETWORK META-ANALYSIS"

Edoardo Pirera [1,*,†], Domenico Di Raimondo [1,†], Lucio D'Anna [2] and Antonino Tuttolomondo [1]

[1] Internal Medicine and Stroke Care Ward, Department of Promoting Health, Maternal-Infant, Excellence and Internal and Specialized Medicine (Promise) G. D'Alessandro, University of Palermo, Piazza delle Cliniche 2, 90127 Palermo, Italy

[2] Department of Stroke and Neuroscience, Charing Cross Hospital, Imperial College London NHS Healthcare Trust, Fulham Palace Rd, London W6 8RF, UK

[†] These two authors equally contributed to the paper.

* Corresponding author: Edoardo Pirera, MD;

Internal Medicine and Stroke Care ward, Department of Promoting Health, Maternal-Infant, Excellence and Internal and Specialized Medicine (Promise) G. D'Alessandro, University of Palermo, Piazza delle Cliniche 2; Palermo, 90127 (Italy);

Supplementary Materials Legend

- **Supplementary Table S1. Search strategy for each database.**
- **Supplementary Table S2. PRISMA-NMA checklist.**
- **Supplementary Table S3. MOOSE Checklist for Meta-analyses of Observational Studies.**
- **Supplementary Table S4. Reason for exclusion at full-text screening.**
- **Supplementary Table S5. Baseline characteristics of study participants.**
- **Supplementary Table S6. Risk of bias assessment according to ROBINS-I.**
- **Supplementary Figure S1. Network geometry for "severe COPD exacerbations".**
- **Supplementary Figure S2. Network geometry for "moderate COPD exacerbations"**
- **Supplementary Figure S3. Summary estimates of primary and secondary outcomes.**
- **Supplementary Figure S4. Diagnostic statistic of NMA – Trace Plot (left) and Gelman-Rubin Plot (right).**
- **Supplementary S1. Results of sensitivity analyses.**

Supplementary Table S1. Search strategy for each database.

**PubMed**	("chronic obstructive lung disease" OR "COPD" OR "chronic obstructive lung disease" OR "chronic obstructive pulmonary disorder" OR "chronic obstructive airway disease") AND (("Glucagon-Like Peptide-1 Receptor Agonists" OR "Glucagon Like Peptide-1 Receptor Agonists" OR "Glucagon Like Peptide 1 Receptor Agonists" OR "Glucagon-Like Peptide 1 Receptor Agonists" OR "GLP1-agonist" OR "GLP-1 agonist" OR "GLP 1 agonist" OR "GLP-1 receptor agonist" OR "GLP-1 ra" OR "GLP1-ra" OR "GLP 1 ra" OR "exenatide" OR "liraglutide" OR "dulaglutide" OR "semaglutide" OR "lixisenatide" OR "tirzepatide") OR ("Sodium Glucose Transporter 2 Inhibitor" OR "Sodium-Glucose Transporter 2 Inhibitor" OR "SGLT-2 Inhibitor" OR "SGLT 2 Inhibitor" OR "SGLT2-I" OR "SGLT2 I" OR "Gliflozin" OR "Dapagliflozin" OR "Empagliflozin" OR "Canagliflozin" OR "Ertugliflozin" OR "Sotagliflozin") OR ("Dipeptidyl Peptidase 4 Inhibitor*" OR "Dipeptidyl Peptidase IV Inhibitor*" OR "Dipeptidyl-Peptidase 4 Inhibitor*" OR "Dipeptidyl-Peptidase IV Inhibitor*" OR "DPP 4 Inhibitor*" OR "DPP IV Inhibitor*" OR "DPP-4 Inhibitor*" OR "DPP-IV Inhibitor*" OR "DPP4 Inhibitor*" OR "gliptin*" OR "vildagliptin" OR "anagliptin" OR "saxagliptin" OR "sitagliptin" OR "alogliptin" OR "linagliptin" OR "evogliptin" OR "gemigliptin" OR "teneligliptin" OR "gosogliptin" OR "trelagliptin" OR "omarigliptin") OR ("Acetohexamide" OR "Carbutamide" OR "Chlorpropamide" OR "Gliclazide" OR "Glyburide" OR "Tolazamide" OR "Tolbutamide" OR "sulfonylurea*"))
**Embase**	('copd'/exp OR copd OR 'chronic obstructive lung disease'/exp OR 'chronic obstructive lung disease' OR 'chronic obstructive pulmonary disorder'/exp OR 'chronic obstructive pulmonary disorder' OR 'chronic obstructive airway disease') AND ('glucagon-like peptide-1 receptor agonists'/exp OR 'glucagon-like peptide-1 receptor agonists' OR 'glucagon like peptide-1 receptor agonists'/exp OR 'glucagon like peptide-1 receptor agonists' OR 'glucagon like peptide 1 receptor agonists'/exp OR 'glucagon like peptide 1 receptor agonists' OR 'glucagon-like peptide 1 receptor agonists'/exp OR 'glucagon-like peptide 1 receptor agonists' OR 'glp1 agonist' OR 'glp-1 agonist'/exp OR 'glp-1 agonist' OR 'glp 1 agonist'/exp OR 'glp 1 agonist' OR 'glp-1 receptor agonist'/exp OR 'glp-1 receptor agonist' OR 'glp-1 ra' OR 'glp1 ra' OR 'glp 1 ra' OR 'exenatide'/exp OR exenatide OR 'liraglutide'/exp OR liraglutide OR 'dulaglutide'/exp OR dulaglutide OR 'semaglutide'/exp OR semaglutide OR 'lixisenatide'/exp OR lixisenatide OR 'tirzepatide'/exp OR tirzepatide OR 'sodium glucose transporter 2 inhibitor' OR 'sodium-glucose transporter 2 inhibitor' OR 'sglt-2 inhibitor' OR 'sglt 2 inhibitor' OR 'sglt2 i' OR 'gliflozin'/exp OR gliflozin OR 'dapagliflozin'/exp OR dapagliflozin OR 'empagliflozin'/exp OR empagliflozin OR 'canagliflozin'/exp OR canagliflozin OR 'ertugliflozin'/exp OR ertugliflozin OR 'sotagliflozin'/exp OR sotagliflozin OR 'dipeptidyl peptidase 4 inhibitor*' OR 'dipeptidyl peptidase iv inhibitor*' OR 'dipeptidyl-peptidase 4 inhibitor*' OR 'dipeptidyl-peptidase iv inhibitor*' OR 'dpp 4 inhibitor*' OR 'dpp iv inhibitor*' OR 'dpp-4 inhibitor*' OR 'dpp-iv inhibitor*' OR 'dpp4 inhibitor*' OR gliptin* OR 'vildagliptin'/exp OR vildagliptin OR 'anagliptin'/exp OR anagliptin OR 'saxagliptin'/exp OR saxagliptin OR 'sitagliptin'/exp OR sitagliptin OR 'alogliptin'/exp OR alogliptin OR 'linagliptin'/exp OR linagliptin OR 'evogliptin'/exp OR evogliptin OR 'gemigliptin'/exp OR gemigliptin OR 'teneligliptin'/exp OR teneligliptin OR 'gosogliptin'/exp OR gosogliptin OR 'trelagliptin'/exp OR trelagliptin OR 'omarigliptin'/exp OR omarigliptin OR 'acetohexamide'/exp OR acetohexamide OR 'carbutamide'/exp OR carbutamide OR 'chlorpropamide'/exp OR chlorpropamide OR 'gliclazide'/exp OR gliclazide OR 'glyburide'/exp OR glyburide OR 'tolazamide'/exp OR tolazamide OR 'tolbutamide'/exp OR tolbutamide OR 'sulfonylurea*')
**Web Of Science**	("chronic obstructive lung disease" OR COPD OR "chronic obstructive lung disease" OR "chronic obstructive pulmonary disorder" OR "chronic obstructive airway disease") AND (("Glucagon-Like Peptide-1 Receptor Agonists" OR "Glucagon Like Peptide-1 Receptor Agonists" OR "Glucagon Like Peptide 1 Receptor Agonists" OR "Glucagon-Like Peptide 1 Receptor Agonists" OR GLP1-agonist OR "GLP-1 agonist" OR "GLP 1 agonist" OR "GLP-1 receptor agonist" OR "GLP-1 ra" OR GLP1-ra OR "GLP 1 ra" OR exenatide OR liraglutide OR dulaglutide OR semaglutide OR lixisenatide OR tirzepatide) OR ("Sodium Glucose Transporter 2 Inhibitor" OR "Sodium-Glucose Transporter 2 Inhibitor" OR "SGLT-2 Inhibitor" OR "SGLT 2 Inhibitor" OR SGLT2-I OR "SGLT2 I" OR Gliflozin OR Dapagliflozin OR Empagliflozin OR Canagliflozin OR Ertugliflozin OR Sotagliflozin) OR ("Dipeptidyl Peptidase 4 Inhibitor*" OR "Dipeptidyl

	Peptidase IV Inhibitor*" OR "Dipeptidyl-Peptidase 4 Inhibitor*" OR "Dipeptidyl-Peptidase IV Inhibitor*" OR "DPP 4 Inhibitor*" OR "DPP IV Inhibitor*" OR "DPP-4 Inhibitor*" OR "DPP-IV Inhibitor*" OR "DPP4 Inhibitor*" OR gliptin* OR vildagliptin OR anagliptin OR saxagliptin OR sitagliptin OR alogliptin OR linagliptin OR evogliptin OR gemigliptin OR teneligliptin OR gosogliptin OR trelagliptin OR omarigliptin) OR (Acetohexamide OR Carbutamide OR Chlorpropamide OR Gliclazide OR Glyburide OR Tolazamide OR Tolbutamide OR sulfonylurea*))
**Central Register of Controlled Trials (CENTRAL) and Cochrane Reviews And clinicaltrial.gov**	("chronic obstructive lung disease" OR "COPD" OR "chronic obstructive lung disease" OR "chronic obstructive pulmonary disorder" OR "chronic obstructive airway disease") AND (("Glucagon-Like Peptide-1 Receptor Agonists" OR "Glucagon Like Peptide-1 Receptor Agonists" OR "Glucagon Like Peptide 1 Receptor Agonists" OR "Glucagon-Like Peptide 1 Receptor Agonists" OR "GLP1-agonist" OR "GLP-1 agonist" OR "GLP 1 agonist" OR "GLP-1 receptor agonist" OR "GLP-1 ra" OR "GLP1-ra" OR "GLP 1 ra" OR "exenatide" OR "liraglutide" OR "dulaglutide" OR "semaglutide" OR "lixisenatide" OR "tirzepatide") OR ("Sodium Glucose Transporter 2 Inhibitor" OR "Sodium-Glucose Transporter 2 Inhibitor" OR "SGLT-2 Inhibitor" OR "SGLT 2 Inhibitor" OR "SGLT2-I" OR "SGLT2 I" OR "Gliflozin" OR "Dapagliflozin" OR "Empagliflozin" OR "Canagliflozin" OR "Ertugliflozin" OR "Sotagliflozin") OR ("Dipeptidyl Peptidase 4 Inhibitor*" OR "Dipeptidyl Peptidase IV Inhibitor*" OR "Dipeptidyl-Peptidase 4 Inhibitor*" OR "Dipeptidyl-Peptidase IV Inhibitor*" OR "DPP 4 Inhibitor*" OR "DPP IV Inhibitor*" OR "DPP-4 Inhibitor*" OR "DPP-IV Inhibitor*" OR "DPP4 Inhibitor*" OR "gliptin*" OR "vildagliptin" OR "anagliptin" OR "saxagliptin" OR "sitagliptin" OR "alogliptin" OR "linagliptin" OR "evogliptin" OR "gemigliptin" OR "teneligliptin" OR "gosogliptin" OR "trelagliptin" OR "omarigliptin") OR ("Acetohexamide" OR "Carbutamide" OR "Chlorpropamide" OR "Gliclazide" OR "Glyburide" OR "Tolazamide" OR "Tolbutamide" OR "sulfonylurea*"))

Supplementary Table S2. PRISMA-NMA checklist [1].

Section/Topic	Item #	Checklist Item	*Reported on Page #*
**TITLE**			
Title	1	Identify the report as a systematic review *incorporating a network meta-analysis (or related form of meta-analysis).*	Page 1
**ABSTRACT**			
Structured summary	2	Provide a structured summary including, as applicable:<br>**Background:** main objectives<br>**Methods:** data sources; study eligibility criteria, participants, and interventions; study appraisal; and *synthesis methods, such as network meta-analysis.*<br>**Results:** number of studies and participants identified; summary estimates with corresponding confidence/credible intervals; *treatment rankings may also be discussed. Authors may choose to summarize pairwise comparisons against a chosen treatment included in their analyses for brevity.*<br>**Discussion/Conclusions:** limitations; conclusions and implications of findings.<br>**Other:** primary source of funding; systematic review registration number with registry name.	Page 1
**INTRODUCTION**			
Rationale	3	Describe the rationale for the review in the context of what is already known*, including mention of why a network meta-analysis has been conducted.*	Page 2-3
Objectives	4	Provide an explicit statement of questions being addressed, with reference to participants, interventions, comparisons, outcomes, and study design (PICOS).	Page 2
**METHODS**			
Protocol and registration	5	Indicate whether a review protocol exists and if and where it can be accessed (e.g., Web address); and, if available, provide registration information, including registration number.	Page 2-4
Eligibility criteria	6	Specify study characteristics (e.g., PICOS, length of follow-up) and report characteristics (e.g., years considered, language, publication status) used as criteria for eligibility, giving rationale. *Clearly describe eligible treatments included in the treatment network, and note whether any have been clustered or merged into the same node (with justification).*	Page 2-4
Information sources	7	Describe all information sources (e.g., databases with dates of coverage, contact with study authors to identify additional studies) in the search and date last searched.	Page 2-4
Search	8	Present full electronic search strategy for at least one database, including any limits used, such that it could be repeated.	Page 2-4
Study selection	9	State the process for selecting studies (i.e., screening, eligibility, included in systematic review, and, if applicable, included in the meta-analysis).	Page 2-4
Data collection process	10	Describe method of data extraction from reports (e.g., piloted forms, independently, in duplicate) and any processes for obtaining and confirming data from	Page 2-4

		investigators.	
Data items	11	List and define all variables for which data were sought (e.g., PICOS, funding sources) and any assumptions and simplifications made.	Page 2-4
**Geometry of the network**	**S1**	Describe methods used to explore the geometry of the treatment network under study and potential biases related to it. This should include how the evidence base has been graphically summarized for presentation, and what characteristics were compiled and used to describe the evidence base to readers.	Page 5;
Risk of bias within individual studies	12	Describe methods used for assessing risk of bias of individual studies (including specification of whether this was done at the study or outcome level), and how this information is to be used in any data synthesis.	Page 3
Summary measures	13	State the principal summary measures (e.g., risk ratio, difference in means). *Also describe the use of additional summary measures assessed, such as treatment rankings and surface under the cumulative ranking curve (SUCRA) values, as well as modified approaches used to present summary findings from meta-analyses.*	Page 3-4
Planned methods of analysis	14	Describe the methods of handling data and combining results of studies for each network meta-analysis. This should include, but not be limited to:<br>• *Handling of multi-arm trials;*<br>• *Selection of variance structure;*<br>• *Selection of prior distributions in Bayesian analyses; and*<br>• *Assessment of model fit.*	Page 3-4; Supplementary S1
**Assessment of Inconsistency**	**S2**	Describe the statistical methods used to evaluate the agreement of direct and indirect evidence in the treatment network(s) studied. Describe efforts taken to address its presence when found.	Page 3-4; Supplementary S1
Risk of bias across studies	15	Specify any assessment of risk of bias that may affect the cumulative evidence (e.g., publication bias, selective reporting within studies).	N/A
Additional analyses	16	Describe methods of additional analyses if done, indicating which were pre-specified. This may include, but not be limited to, the following:<br>• Sensitivity or subgroup analyses;<br>• Meta-regression analyses;<br>• *Alternative formulations of the treatment network; and*<br>• *Use of alternative prior distributions for Bayesian analyses (if applicable).*	Page 3-4; Supplementary S1
**RESULTS†**			
Study selection	17	Give numbers of studies screened, assessed for eligibility, and included in the review, with reasons for exclusions at each stage, ideally with a flow diagram.	Page 4
**Presentation of network structure**	**S3**	Provide a network graph of the included studies to enable visualization of the geometry of the treatment network.	Figure 2; Figures S1-S2;

**Summary of network geometry**	**S4**	Provide a brief overview of characteristics of the treatment network. This may include commentary on the abundance of trials and randomized patients for the different interventions and pairwise comparisons in the network, gaps of evidence in the treatment network, and potential biases reflected by the network structure.	Page 5
Study characteristics	18	For each study, present characteristics for which data were extracted (e.g., study size, PICOS, follow-up period) and provide the citations.	Page 4; Table 1
Risk of bias within studies	19	Present data on risk of bias of each study and, if available, any outcome level assessment.	Supplementary Table S5
Results of individual studies	20	For all outcomes considered (benefits or harms), present, for each study: 1) simple summary data for each intervention group, and 2) effect estimates and confidence intervals. *Modified approaches may be needed to deal with information from larger networks.*	Supplementary S1
Synthesis of results	21	Present results of each meta-analysis done, including confidence/credible intervals. *In larger networks, authors may focus on comparisons versus a particular comparator (e.g. placebo or standard care), with full findings presented in an appendix. League tables and forest plots may be considered to summarize pairwise comparisons.* If additional summary measures were explored (such as treatment rankings), these should also be presented.	Page 5-7; Figure 2; Supplementary Figure S3
**Exploration for inconsistency**	**S5**	Describe results from investigations of inconsistency. This may include such information as measures of model fit to compare consistency and inconsistency models, *P* values from statistical tests, or summary of inconsistency estimates from different parts of the treatment network.	Supplementary S1
Risk of bias across studies	22	Present results of any assessment of risk of bias across studies for the evidence base being studied.	Supplementary Table S5
Results of additional analyses	23	Give results of additional analyses, if done (e.g., sensitivity or subgroup analyses, meta-regression analyses, *alternative network geometries studied, alternative choice of prior distributions for Bayesian analyses,* and so forth).	Supplementary S1
**DISCUSSION**			
Summary of evidence	24	Summarize the main findings, including the strength of evidence for each main outcome; consider their relevance to key groups (e.g., healthcare providers, users, and policy-makers).	Page 7-10
Limitations	25	Discuss limitations at study and outcome level (e.g., risk of bias), and at review level (e.g., incomplete retrieval of identified research, reporting bias). *Comment on the validity of the assumptions, such as transitivity and consistency. Comment on any concerns regarding network geometry (e.g., avoidance of certain comparisons).*	Page 7-10
Conclusions	26	Provide a general interpretation of the results in the context of other evidence, and implications for future research.	Page 10
**FUNDING**			
Funding	27	Describe sources of funding for the systematic review and other support (e.g., supply of data); role of funders for the systematic review. This should also include information regarding whether funding has been received from manufacturers of treatments in the network and/or whether some of the authors are content experts with professional conflicts of interest that could affect use of treatments in the	Page 10

	network.	

PICOS = population, intervention, comparators, outcomes, study design.

* Text in italics indicates wording specific to reporting of network meta-analyses that has been added to guidance from the PRISMA statement.

† Authors may wish to plan for use of appendices to present all relevant information in full detail for items in this section.

N/A: Not applicable.

Supplementary Table S3. MOOSE Checklist for Meta-analyses of Observational Studies [2]

Item No	Recommendation	Reported on Page No
Reporting of background should include		
1	Problem definition	Page 2
2	Hypothesis statement	Page 2
3	Description of study outcome(s)	Page 3
4	Type of exposure or intervention used	Page 3
5	Type of study designs used	Page 3
6	Study population	Page 3
Reporting of search strategy should include		
7	Qualifications of searchers (eg, librarians and investigators)	Page 3
8	Search strategy, including time period included in the synthesis and key words	Page 2-3
9	Effort to include all available studies, including contact with authors	Page 2-3
10	Databases and registries searched	Page 2
11	Search software used, name and version, including special features used (eg, explosion)	N/A
12	Use of hand searching (eg, reference lists of obtained articles)	N/A
13	List of citations located and those excluded, including justification	N/A
14	Method of addressing articles published in languages other than English	Page 2-3
15	Method of handling abstracts and unpublished studies	Page 3
16	Description of any contact with authors	N/A
Reporting of methods should include		
17	Description of relevance or appropriateness of studies assembled for assessing the hypothesis to be tested	Page 4
18	Rationale for the selection and coding of data (eg, sound clinical principles or convenience)	N/A
19	Documentation of how data were classified and coded (eg, multiple raters, blinding and interrater reliability)	Page 4
20	Assessment of confounding (eg, comparability of cases and controls in studies where appropriate)	N/A
21	Assessment of study quality, including blinding of quality assessors, stratification or regression on possible predictors of study results	N/A
22	Assessment of heterogeneity	Page 3-4
23	Description of statistical methods (eg, complete description of fixed or random effects models, justification of whether the chosen models account for predictors of study results, dose-response models, or cumulative meta-analysis) in sufficient detail to be replicated	Page 3-4
24	Provision of appropriate tables and graphics	Page 3-4
Reporting of results should include		
25	Graphic summarizing individual study estimates and overall estimate	Supplementary S1
26	Table giving descriptive information for each study included	Table1
27	Results of sensitivity testing (eg, subgroup analysis)	Supplementary S1

28	Indication of statistical uncertainty of findings	Page 4-7
Reporting of discussion should include		
29	Quantitative assessment of bias (e.g., publication bias)	N/A
30	Justification for exclusion (e.g., exclusion of non-English language citations)	Figure 1
31	Assessment of quality of included studies	Supplementary Table S5
Reporting of conclusions should include		
32	Consideration of alternative explanations for observed results	Page 7-10
33	Generalization of the conclusions (i.e., appropriate for the data presented and within the domain of the literature review)	Page 7-10
34	Guidelines for future research	Page 7-10
35	Disclosure of funding source	Page 10

Supplementary Table S4. Reason for exclusion at full-text screening.

**"Title", *First Author and year* [DOI]**	**Reason for exclusion**
"The Potential of Antidiabetic Medications in the Prevention of Acute Exacerbations of Chronic Obstructive Pulmonary Disease in Subjects with Type 2 Diabetes Mellitus", *Panou T. 2025* [https://doi.org/10.1055/a-2567-7542]	Review Article
"Association of novel antihyperglycaemic drugs versus metformin with COPD exacerbations", *Kimura Y. 2025* [https://doi.org/10.1183/23120541.00757-2024]	Different active comparator
"The possible effect of anti-diabetic agents GLP-1RA and SGLT-2i on the respiratory system function, *Kantreva K. 2025* [https://doi.org/10.1007/s12020-024-04033-6]	Review Article
"Use of Sodium-glucose cotransporter 2 (SGLT 2) inhibitor is associated with reduced emergency room visits and hospitalizations in patients with Chronic obstructive pulmonary disease (COPD) and type 2 Diabetes Mellitus", *Geetha HS 2024* [https://doi.org/10.1016/j.rmed.2024.107819]	No active comparator
"The role of Sodium-Glucose Transporter-2 Inhibitors (SGLT-2i) in preventing chronic obstructive disease exacerbation in patients with diabetes and COPD: An electronic health database analysis", *Gupta S. 2024* [https://doi.org/10.1016/j.hrtlng.2024.07.003]	No active comparator
"Association of DPP-4 inhibitors with respiratory and cardiovascular complications in patients with COPD: a nationwide cohort study", *Yen, F. S. 2025* [https://doi.org/10.1183/23120541.00919-2024]	Transitivity assumption not met
"Sulfonylurea Use in Patients with Type 2 Diabetes and COPD: A Nationwide Population-Based Cohort Study", *Yen F. S. 2022* [10.3390/ijerph192215013]	Different active comparator
"SGLT-2 Inhibitor Use and Cause-Specific Hospitalization Rates: An Outcome-Wide Study to Identify Novel Associations of SGLT-2 Inhibitors", *Tan G.S.Q. 2024* [https://doi.org/10.1002/cpt.3194]	Transitivity assumption not met
"Combination therapies with thiazolidinediones are associated with a lower risk of acute exacerbations in new-onset COPD patients with advanced diabetic mellitus: a cohort-based case-control study", *Chen K. Y. 2021* [https://doi.org/10.1186/s12890-021-01505-7]	No active comparator
"Use of antidiabetic medications and risk of chronic obstructive pulmonary disease exacerbation requiring hospitalization: a disease risk score-matched nested case-control study", *Wang M.T. 2020* [https://doi.org/10.1186/s12931-020-01547-1]	No active comparator
"A Daunting and Challenging Task to Prove the Effectiveness of Reducing Acute Exacerbation in COPD Patients with Type 2 Diabetes by GLP-1 Receptor Agonists", *Li Q. 2023* [https://doi.org/10.1164/rccm.202309-1671LE]	Commentary Article

Supplementary Table S5. Baseline characteristics of study participants.

Author, year [reference]	***T2DM Therapy***	***COPD Therapy***	***Heart Failure***	***Arterial Hypertension***	***Chronic Coronary Syndrome***	***Cerebrovascular disease***	***Chronic Kydney Disease***	***Arrhythmia***	***Dyslipidemia***
**Albogami, 2021** [3]	*Metformin*<br>DPP4i: 4,2;<br>GLP-1RA: 4,3;<br><br>*Sulfonylurea*<br>DPP4i: 4,2;<br>GLP-1RA: 4,3;<br><br>*Glitazone*<br>DPP4i: 2,9;<br>GLP-1RA: 2,9;<br><br>*SGLT2i*<br>DPP4i: 1,5;<br>GLP-1RA: 1,4;	*ICS/LABA/LAMA*<br>DPP4i: N/A;<br>GLP-1RA: N/A;<br><br>*LTOT*<br>DPP4i: 6,1;<br>GLP-1RA: 6,1;<br><br>*OCS*<br>DPP4i: 2;<br>GLP-1RA: 1,9;	DPP4i: 5,4;<br>GLP-1RA: 5,3;	DPP4i: 70,5;<br>GLP-1RA: 70;	DPP4i: 15,5;<br>GLP-1RA: 15,2;	DPP4i: 2,4;<br>GLP-1RA: 2,3;	N/A	DPP4i: 7,7;<br>GLP-1RA: 7,5;	DPP4i: 67,8;<br>GLP-1RA: 67,5;
**Pradhan, 2022** [4]	*Metformin*<br>GLP-1RA: 83,1;<br>DPP4i: 84,8;<br>SGLT2i: 89,6;<br>Sulfonylurea: 82,1;<br><br>*Thiazolidinedione*<br>GLP-1RA: 10,6;<br>DPP4i: 6,2;<br>SGLT2i: 4,3;<br>Sulfonylurea: 7,6;<br><br>*α-glucosidase inhibitor*<br>GLP-1RA: 0,9;<br>DPP4i: 0,2;<br>SGLT2i: 0,2;<br>Sulfonylurea: 0,2;<br><br>*SGLT2i*<br>GLP-1RA: 9,3;<br>DPP4i: 2,8;<br>Sulfonylurea: 0,9;	*GLP-1RA*<br>ICS/LABA/LAMA: N/A;<br>OCS: 36,6;<br><br>*DPP4i*<br>ICS/LABA/LAMA: N/A;<br>OCS: 36,3;<br><br>*SGLT2i*<br>ICS/LABA/LAMA: N/A;<br>OCS: 34,9;<br><br>*Sulfonylurea*<br>ICS/LABA/LAMA: N/A;<br>OCS: 34,9;	GLP-1RA: 19,3;<br>DPP4i: 20,4;<br>SGLT2i: 12,1;<br>Sulfonylurea: 18,8;	GLP-1RA: 85,1;<br>DPP4i: 83,3;<br>SGLT2i: 80,9;<br>Sulfonylurea: 78,3;	GLP-1RA: 16,8;<br>DPP4i: 15,5;<br>SGLT2i: 13,8;<br>Sulfonylurea: 4,8;	GLP-1RA: 8,1;<br>DPP4i: 10,2;<br>SGLT2i: 6,8;<br>Sulfonylurea: 8,7;	GLP-1RA: 24,3;<br>DPP4i:29,8;<br>SGLT2i: 12,8;<br>Sulfonylurea: 25,0;	GLP-1RA: 20,4;<br>DPP4i: 27,9;<br>SGLT2i: 18,5;<br>Sulfonylurea: 23,4;	GLP-1RA: 55,1;<br>DPP4i: 51,2;<br>SGLT2i: 51,0;<br>Sulfonylurea: 44,7;

	Insulin<br>GLP-1RA: 53,6;<br>DPP4i: 11,6;<br>SGLT2i: 25,2;<br>Sulfonylurea: 5,7;<br>*GLP-1RA*<br>SGLT2i: 11,4;<br>*DPP4i*<br>SGLT2i: 32,9;								
**Foer, 2023** [5]	*Metformin*<br>DPP4i: 47,69;<br>GLP-1RA: 54,57;<br>SGLT2i: 50,71;<br>Sulfonylurea: 34,81;	*DPP4i*<br>ICS/LABA/LAMA: 1,92;<br>*GLP-1RA*<br>ICS-LABA-LAMA: 3,66;<br>*SGLT2i*<br>ICS-LABA-LAMA: 5,67;<br>*Sulfonylurea*<br>ICS-LABA-LAMA: 1,28;	DPP4i: 35,38;<br>GLP-1RA: 35,98;<br>SGLT2i: 57,79;<br>Sulfonylurea: 7,96;	DPP4i: 66,15;<br>GLP-1RA: 80,79;<br>SGLT2i: 89,80;<br>Sulfonylurea: 1,48;	N/A	N/A	N/A	N/A	N/A
**Au, 2023** [6]	*Metformin*<br>DPP4i: 81,8;<br>SGLT2i: 83,5;<br>*Sulfonylurea*<br>DPP4i: 62,1;<br>SGLT2i: 62,7;<br>*GLP-1RA*<br>DPP4i: 0;<br>SGLT2i: 0;<br>*Thiazolidinedione*<br>DPP4i: 7,0;<br>SGLT2i: 7,3;<br>*α-glucosidase inhibitor*<br>DPP4i: 1,6;<br>SGLT2i: 2,4;<br>*Insulin*<br>DPP4i: 35,6;<br>SGLT2i: 35,4;	*ICS, LABA/ICS, ICS/LABA/LAMA*<br>DPP4i: 27,0;<br>SGLT2i: 28,9;	DPP4i: 0;<br>SGLT2i: 0;	DPP4i: 25,6;<br>SGLT2i: 23,6;	DPP4i: 12,6;<br>SGLT2i: 12,6;	DPP4i: 4,3;<br>SGLT2i: 4,5;	DPP4i: 12,4;<br>SGLT2i: 12,6;	DPP4i: 3,3;<br>SGLT2i: 3,7;	DPP4i: 14,8;<br>SGLT2i: 14,2;

**See, 2024** [7]	*Biguanide*<br>GLP-1RA: 46,9;<br>DPP4i: 47,0;<br><br>*Sulfonylurea*<br>GLP-1RA: 21,8;<br>DPP4i: 21,8;<br><br>*Thiazolidinedione*<br>GLP-1RA: 3,7;<br>DPP4i: 3,4;<br><br>*α-glucosidase inhibitor*<br>GLP-1RA: 0,6;<br>DPP4i: 0,6;<br><br>*Insulin*<br>GLP-1RA: 56,0;<br>DPP4i: 56,0;<br><br>*SGLT2i*<br>GLP-1RA: 14,5;<br>DPP4i: 14,5;	*ICS/LABA/LAMA*<br>GLP-1RA: 100;<br>DPP4i: 100;<br><br>*OCS*<br>GLP-1RA: 7,8;<br>DPP4i: 8,1;<br><br>*LTOT*<br>GLP-1RA: 18,2;<br>DPP4i: 18,3;	N/A	GLP-1RA: 76,9;<br>DPP4i: 76,9;	GLP-1RA: 45,3;<br>DPP4i: 44,2;	N/A	GLP-1RA: 31,9;<br>DPP4i: 31,1;	N/A	GLP-1RA: 71,7;<br>DPP4i: 71,0;
**Yen, 2024** [8]	*Metformin*<br>SGLT2i: 90,82;<br>GLP-1RA: 90,43;<br><br>*Sulfonylurea*<br>SGLT2i: 66,77;<br>GLP-1RA: 68,17;<br><br>*Thiazolidinedione*<br>SGLT2i: 35,15;<br>GLP-1RA: 35,20;<br><br>*Insulin*<br>SGLT2i: 60,80;<br>GLP-1RA: 61,72;<br><br>*α-glucosidase inhibitor*<br>SGLT2i: 23,85;<br>GLP-1RA: 25,06;	*ICS/LABA/LAMA*<br>SGLT2i: N/A;<br>GLP-1RA: N/A;<br><br>*OCS*<br>SGLT2i: 44,91;<br>GLP-1RA: 47,54;	N/A	SGLT2i: 79,18;<br>GLP-1RA: 79,56;	SGLT2i: 26,37;<br>GLP-1RA: 26,14;	SGLT2i: 7,06;<br>GLP-1RA: 6,82;	SGLT2i: 24,21;<br>GLP-1RA: 24,63;	N/A	SGLT2i: 88,48;<br>GLP-1RA: 87,82;

**Chang, 2025** [9]	N/A	*ICS/LABA/LAMA*<br>DPP4i: 36,46;<br>SGLT2i: 39,36;<br><br>*OCS*<br>DPP4i: 74,03;<br>SGLT2i: 40,96;	DPP4i: 7,73;<br>SGLT2i: 17,55;	DPP4i: 65,75;<br>SGLT2i: 71,81;	DPP4i: 20,44;<br>SGLT2i: 40,43;	DPP4i: 4,42;<br>SGLT2i: 2,13;	N/A	*Atrial Fibrillation*<br>DPP4i: 4,42;<br>SGLT2i: 17,55;	DPP4i: 32,04;<br>SGLT2i: 40,96;
**Ray, 2025** [10]	*Metformin*<br>SGLT2i: 49,5;<br>GLP-1RA: 44,6;<br>DPP4i: 49,6;<br><br>*Sulfonylurea*<br>SGLT2i: 36,0;<br>GLP-1RA: 35,1;<br>DPP4i: 35,7;<br><br>*Thiazolidinedione*<br>GLP-1RA: 6,1;<br><br>*Insulin*<br>SGLT2i: 38,4;<br>GLP-1RA: 48,8;<br>DPP4i: 37,9;<br><br>*SGLT2i*<br>GLP-1RA: 9,5;<br>DPP4i: 9,8;<br><br>*GLP-1RA*<br>SGLT2i: 11,1;<br>DPP4i: 10,3;	*ICS/LABA/LAMA*<br>SGLT2i: 5,7;<br>GLP-1RA: 5,3;<br>DPP4i: 5,8;<br><br>*LTOT*<br>SGLT2i: 12,4;<br>GLP-1RA: 11,2;<br>DPP4i: 12,6;<br><br>*OCS*<br>SGLT2i: 46,2;<br>GLP-1RA: 45,8;<br>DPP4i: 45,8;	SGLT2i: 44,1;<br>GLP-1RA: 44,3;<br>DPP4i: 43,7;	SGLT2i: 95,2;<br>GLP-1RA: 95,5;<br>DPP4i: 95,2;	SGLT2i: 52,8;<br>GLP-1RA: 51,2;<br>DPP4i: 52,4;	SGLT2i: 21,9;<br>GLP-1RA: 21,0;<br>DPP4i: 21,6;	*Stage 1-2*<br>SGLT2i: 5,5;<br>GLP-1RA: 5,5;<br>DPP4i: 5,5;<br><br>*Stage 3-4*<br>SGLT2i: 22,7;<br>GLP-1RA: 27,1;<br>DPP4i: 22,1;<br><br>*Unspecified*<br>SGLT2i: 15,5;<br>GLP-1RA: 19,0;<br>DPP4i: 15,3;	*Atrial Fibrillation*<br>SGLT2i: 27,4;<br>GLP-1RA: 25,5;<br>DPP4i: 26,9;<br><br>*Arrhythmias (excluding atrial fibrillation)*<br>SGLT2i: 19,8;<br>GLP-1RA: 18,3;<br>DPP4i: 19,7;	SGLT2i: 88,7;<br>GLP-1RA: 88,8;<br>DPP4i: 88,5;
**Yen, 2025** [11]	*Metformin*<br>SGLT2i: 93,8;<br>DPP4i: 94,0;<br>Sulfonylurea: 92,6;<br><br>*Sulfonylurea*<br>SGLT2i: 69,9;<br>DPP4i: 68,3;	*ICS/LABA/LAMA*<br>SGLT2i: 16,1;<br>DPP4i: 14,2;<br>Sulfonylurea: 15,1;<br><br>*OCS*<br>SGLT2i: 94,5;<br>DPP4i: 94,5;<br>Sulfonylurea: 94,5;	SGLT2i: 4,4;<br>DPP4i: 4,0;<br>Sulfonylurea: 5,4;	SGLT2i: 75,2;<br>DPP4i: 76,5;<br>Sulfonylurea: 78,5;	SGLT2i: 20,8;<br>DPP4i: 18,3;<br>Sulfonylurea: 20,2;	SGLT2i: 7,9;<br>DPP4i: 7,0;<br>Sulfonylurea: 7,0;	SGLT2i: 15,9;<br>DPP4i: 14,4;<br>Sulfonylurea: 17,3;	SGLT2i: 11,9;<br>DPP4i: 7,3;<br>Sulfonylurea: 11,2;	SGLT2i: 81,8;<br>DPP4i: 82,9;<br>Sulfonylurea: 81,1;

	Thiazolidinedione<br>SGLT2i: 24,3;<br>DPP4i: 24,1;<br>Sulfonylurea: 19,2;<br><br>*Insulin*<br>SGLT2i: 49,3;<br>DPP4i: 47,3;<br>Sulfonylurea: 50,3;<br><br>*α-glucosidase inhibitor*<br>SGLT2i: 22,3;<br>DPP4i: 20,6;<br>Sulfonylurea: 26,6;<br><br>*GLP-1RA*<br>SGLT2i: 12,2;<br>DPP4i: 11,7;<br>Sulfonylurea: 15,0;<br><br>*DPP4i*<br>SGLT2i: 55,1;<br>Sulfonylurea: 50,6;								

Legend: Data are reported as percentage (%), unless otherwise stated. Efforts were made to report all available data and where data were unavailable, the most informative metrics were provided.

Abbreviations: AECOPD: acute exacerbation of chronic obstructive pulmonary disease; COPD: Chronic obstructive pulmonary disease; DPP4i: dipeptidyl peptidase-4 inhibitor; FEV_1: forced expiratory volume in one second; GOLD: global initiative for chronic obstructive lung disease; GLP-1RA: glucagon-like peptide-1 receptor agonist; ICS: inhaled corticosteroid; LABA: long-acting beta-agonist; LAMA: long-acting muscarinic antagonist; LTOT: long-term oxygen therapy; n: number; N/A: not available/reported; OCS: oral corticosteroid; SD: standard deviation; SGLT2i: sodium-glucose cotransporter-2 inhibitor; T2DM: type 2 diabetes mellitus;

Supplementary Table S6. Risk of bias assessment according to ROBINS-I [12]

Author, year and reference	Bias due to confounding	Bias in selection of participants	Bias in classification of interventions	Bias due to deviations from intended interventions	Bias due to missing data	Bias in measurement of outcomes	Bias in selection of the reported result	Overall judgement
**Albogami, 2021** [3]	Low risk	Low risk	Low risk	Low risk	Low risk	Low risk	Low risk	Low risk except for concerns about uncontrolled confounding
**Pradhan, 2022** [4]	Low risk	Low risk	Low risk	Low risk	Low risk	Low risk	Low risk	Low risk except for concerns about uncontrolled confounding
**Foer, 2023** [5]	Moderate risk[a]	Low risk	Low risk	Low risk	Low risk	Low risk	Low risk	Moderate risk
**Au, 2023** [6]	Low risk	Low risk	Low risk	Low risk	Low risk	Low risk	Low risk	Low risk except for concerns about uncontrolled confounding
**See, 2024** [7]	Low risk	Low risk	Low risk	Low risk	Low risk	Low risk	Low risk	Low risk except for concerns about uncontrolled confounding
**Yen, 2024** [8]	Low risk	Low risk	Low risk	Low risk	Low risk	Low risk	Low risk	Low risk except for concerns about uncontrolled confounding
**Chang, 2025** [9]	Moderate risk[a]	Low risk	Low risk	Low risk	Low risk	Low risk	Low risk	Moderate risk
**Ray, 2025** [10]	Low risk	Low risk	Low risk	Low risk	Low risk	Low risk	Low risk	Low risk except for concerns about uncontrolled confounding
**Yen, 2025** [11]	Low risk	Low risk	Low risk	Low risk	Low risk	Low risk	Low risk	Low risk except for concerns about uncontrolled confounding

[a] *Foer 2023* [5] and *Chang 2025* [9] were judged at moderate risk as they did not employ matching techniques to control for baseline confounders.

Supplementary Figure S1. Network geometry for “severe COPD exacerbations”.

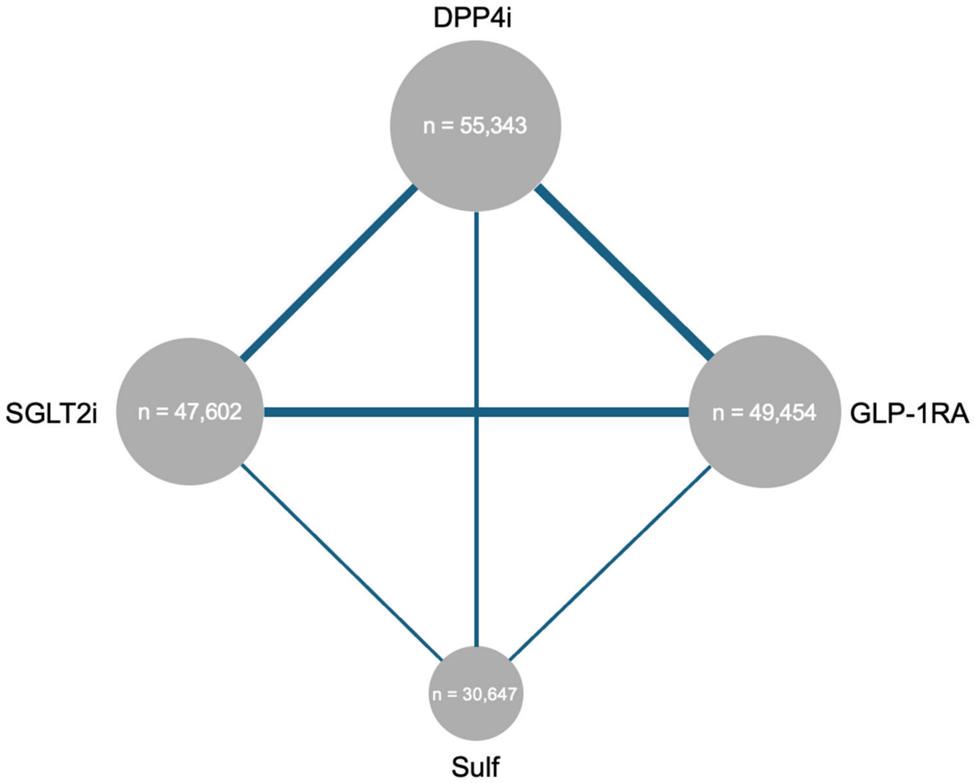

Supplementary Figure S2. Network geometry for “moderate COPD exacerbations”.

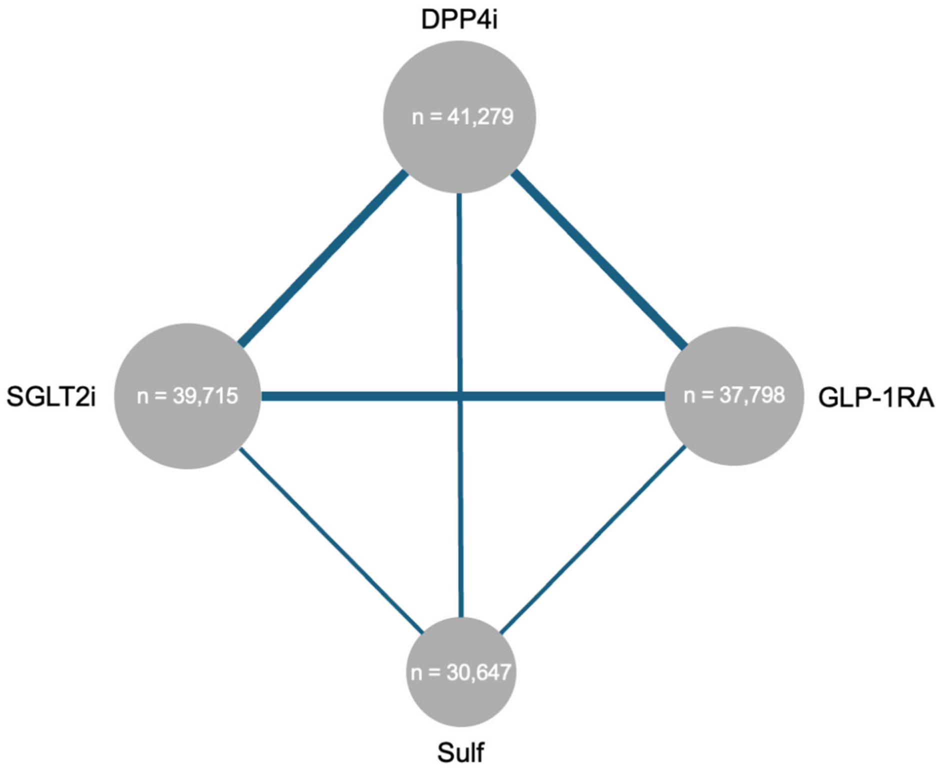

Abbreviations: DPP4i: dipeptidyl peptidase-4 inhibitor; GLP-1RA: glucagon-like peptide-1 receptor agonist; SGLT2i: sodium-glucose cotransporter-2 inhibitor; Sulf: sulfonylurea;

Supplementary Figure S3. Summary estimates of primary and secondary outcomes.

Pairwise comparisons among SGLT2i, GLP-1 RA, DPP4i and sulfonylureas are displayed through league tables. A league table in NMA represents a matrix presentation of all possible pairwise comparisons between interventions within a network. Each cell contains the relative treatment effect of the row treatment versus the column treatment.

- **Supplementary Figure S3.1. Summary estimates for the outcome "moderate or severe exacerbation of COPD"**

**DPP4i**	**0.83 (0.77, 0.88)**	**0.8 (0.75, 0.86)**	**1.26 (1.17, 1.36)**
**1.21 (1.14, 1.3)**	**GLP-1 RA**	0.97 (0.91, 1.06)	**1.52 (1.41, 1.67)**
**1.25 (1.16, 1.33)**	1.03 (0.94, 1.1)	**SGLT2i**	**1.57 (1.44, 1.7)**
**0.79 (0.74, 0.86)**	**0.66 (0.6, 0.71)**	**0.64 (0.59, 0.69)**	**Sulf**

- **Supplementary Figure S3.2. Summary estimates for the secondary outcome "moderate exacerbation of COPD"**

**DPP4i**	**0.88 (0.77, 0.98)**	**0.85 (0.77, 0.95)**	**1.17 (1.04, 1.33)**
**1.14 (1.02, 1.3)**	**GLP1RA**	0.97 (0.87, 1.1)	**1.33 (1.18, 1.54)**
**1.17 (1.06, 1.31)**	1.03 (0.91, 1.15)	**SGLT2i**	**1.37 (1.22, 1.56)**
**0.86 (0.75, 0.96)**	**0.75 (0.65, 0.85)**	**0.73 (0.64, 0.82)**	**Sulf**

- **Supplementary Figure S3.3. Summary estimates for the secondary outcome "severe exacerbation of COPD"**

**DPP4i**	**0.63 (0.47, 0.83)**	**0.64 (0.50, 0.82)**	1.21 (0.88, 1.63)
**1.59 (1.20, 2.15)**	**GLP1RA**	1.02 (0.77, 1.36)	**1.92 (1.40, 2.65)**
**1.56 (1.22, 2.01)**	0.98 (0.74, 1.29)	**SGLT2i**	**1.89 (1.41, 2.51)**
0.83 (0.62, 1.13)	**0.52 (0.38, 0.71)**	**0.53 (0.40, 0.71)**	**Sulf**

Data are reported as risk ratios and 95% credible interval. Data in bold are statistically significant. Abbreviations: DPP4i: dipeptidyl peptidase-4 inhibitor; GLP1RA: glucagon-like peptide-1 receptor agonist; SGLT2i: sodium-glucose cotransporter-2 inhibitor; Sulf: sulfonylurea;

Supplementary Figure S4. Diagnostic statistic of NMA – Trace Plot (left) and Gelman-Rubin Plot (right).

We checked convergence of the fitted models under the assumption of evidence consistency using the trace plots (left) and Gelman-Rubin plots (right). Trace plots represent a visual diagnostic for evaluating convergence properties of Markov chain Monte Carlo (MCMC) algorithms in Bayesian inference and display the sequential values of parameter samples against iteration number for each chain in the simulation. The Gelman-Rubin statistic quantifies convergence by analysing the variance structure within and between chains. As shown below, trace plots and Gelman-Rubin plots of our models showed that each MCMC chain converged well.

- **Supplementary Figure S4.1. Diagnostic statistic of NMA for the outcome "moderate or severe exacerbation of COPD"**

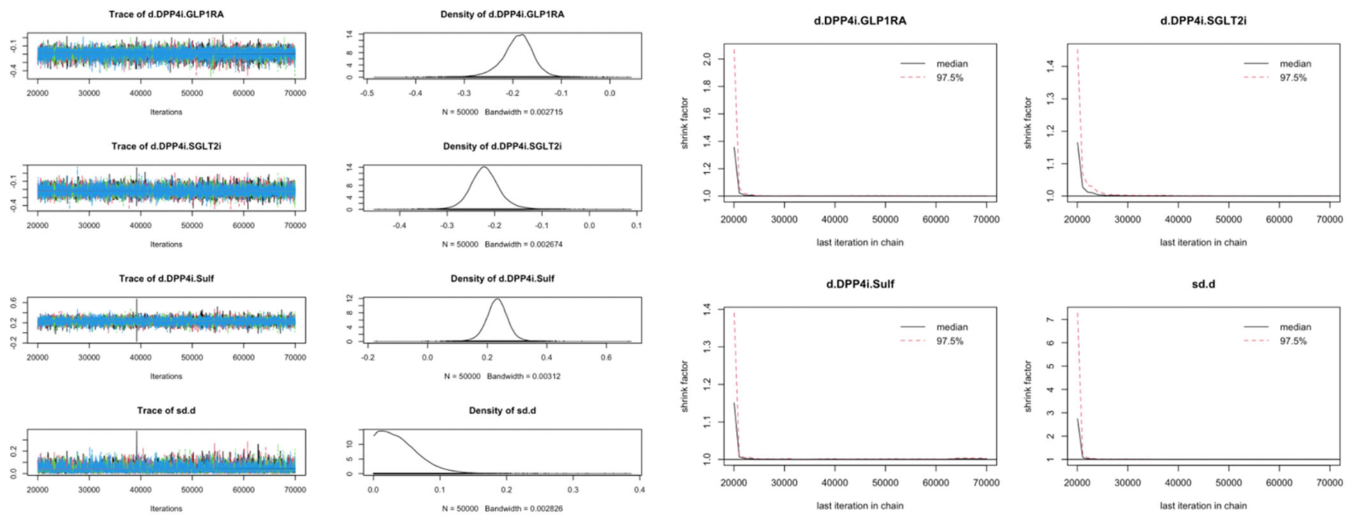

- **Supplementary Figure S4.2. Diagnostic statistic of NMA for the outcome "severe exacerbation of COPD"**

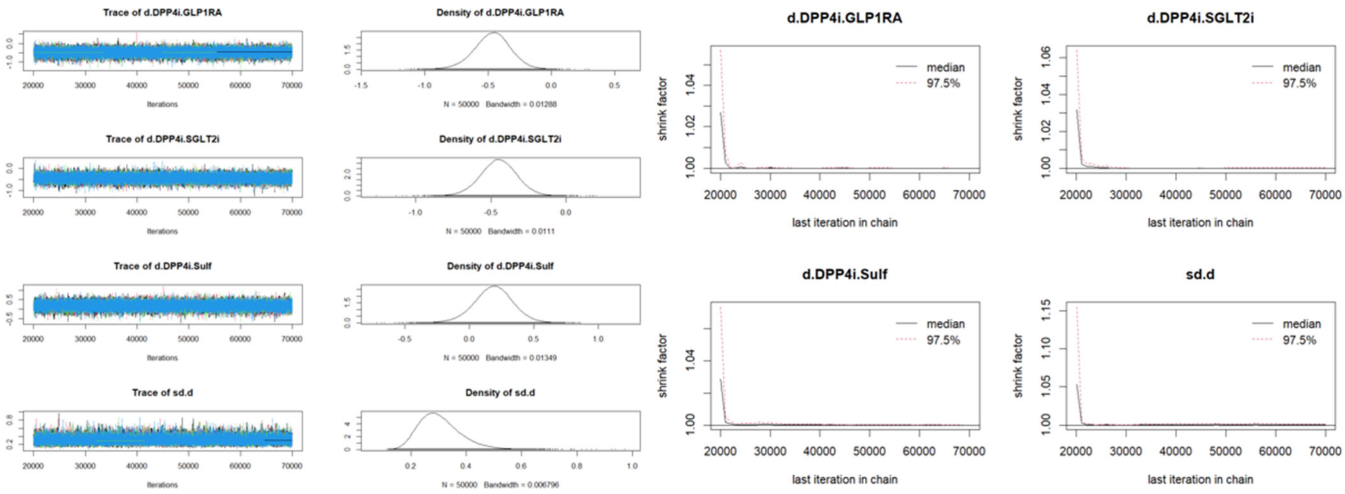

- **Supplementary Figure S4.3. Diagnostic statistic of NMA for the outcome "moderate exacerbation of COPD"**

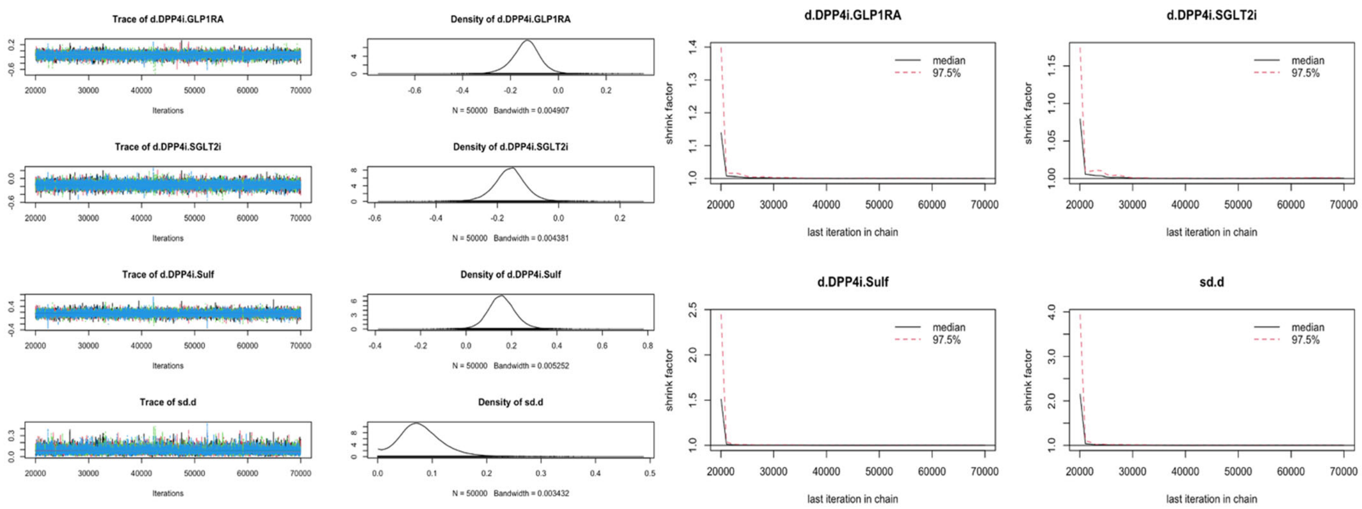

Supplementary S1. Results of sensitivity analyses

To assess the robustness of our network meta-analysis (NMA) model, we performed several sensitivity analyses: (1) a frequentist approach; (2) a node-splitting approach; (3) a comparison of Bayesian model fit with and without the assumption of evidence inconsistency; (4) a comparison of Bayesian model fit using different prior distributions; (5) NMA using adjusted effect estimates reported in the included studies; (6) NMA including only studies judged at "low risk" of bias according to ROBINS-I.

1. NMA using a frequentist approach

1.1. Summary estimates for the outcome "moderate or severe exacerbation of COPD"

**DPP4i**	**0.83 (0.81, 0.87)**	**0.80 (0.77, 0.83)**	**1.26 (1.20, 1.32)**
**1.20 (1.15, 1.24)**	**GLP1RA**	**0.96 (0.92, 0.99)**	**1.51 (1.43, 1.59)**
**1.25 (1.20, 1.30)**	**1.04 (1.002, 1.09)**	**SGLT2i**	**1.59 (1.49, 1.67)**
**0.79 (0.76, 0.83)**	**0.66 (0.63, 0.70)**	**0.63 (0.60, 0.67)**	**Sulf**

1.2. Summary estimates for the outcome "moderate exacerbation of COPD"

**DPP4i**	**0.87 (0.79, 0.96)**	**0.85 (0.78, 0.93)**	**1.17 (1.05, 1.30)**
**1.15 (1.04, 1.27)**	**GLP1RA**	0.98 (0.88, 1.09)	**1.35 (1.19, 1.51)**
**1.17 (1.07, 1.28)**	1.02 (0.92, 1.13)	**SGLT2i**	**1.37 (1.23, 1.54)**
**0.85 (0.77, 0.95)**	**0.74 (0.66, 0.84)**	**0.73 (0.65, 0.81)**	**Sulf**

1.3. Summary estimates for the outcome "severe exacerbation of COPD"

**DPP4i**	**0.63 (0.49, 0.81)**	**0.64 (0.51, 0.80)**	1.20 (0.92, 1.59)
**1.58 (1.23, 2.04)**	**GLP1RA**	1.02 (0.79, 1.30)	**1.92 (1.45, 2.44)**
**1.56 (1.25, 1.94)**	0.98 (0.77, 1.26)	**SGLT2i**	**1.88 (1.45, 2.44)**
0.83 (0.63, 1.08)	**0.52 (0.40, 0.69)**	**0.53 (0.41, 0.69)**	**Sulf**

Data are reported as risk ratios and 95% confidence interval. Data in bold are statistically significant. Abbreviations: DPP4i: dipeptidyl peptidase-4 inhibitor; GLP1RA: glucagon-like peptide-1 receptor agonist; SGLT2i: sodium-glucose cotransporter-2 inhibitor; Sulf: sulfonylurea;

2. Summary estimates of the primary and secondary outcomes according to direct, indirect and network evidence of the NMA

Node-splitting is a statistical approach used to assess the consistency assumption in network meta-analysis by comparing direct and indirect evidence for each treatment comparison. The node-splitting graph presents these comparisons in a structured visual format. In the node-splitting graph, each comparison (e.g., A vs. B) is "split" into three estimates: direct evidence (from head-to-head trial of A vs. B), indirect evidence (derived from trials comparing A and B to other treatments), and the combined network estimate. The graph typically displays: (1) Point estimates and credible intervals for direct, indirect, and network evidence; (2) *p*-values for the difference between direct and indirect evidence. Substantial divergence (typically indicated by *p*-values<0.05) suggests potential inconsistency.

Node-splitting analysis showed neglectable evidence of inconsistency for all fitted models, except for the comparison of sulfonylurea and DPP4i for the secondary outcome "severe exacerbation" (*p*=0.0038, Supplementary S1-2.2)

2.1. Summary estimates of the outcome “moderate or severe exacerbation of COPD” according to direct, indirect and network evidence of the NMA

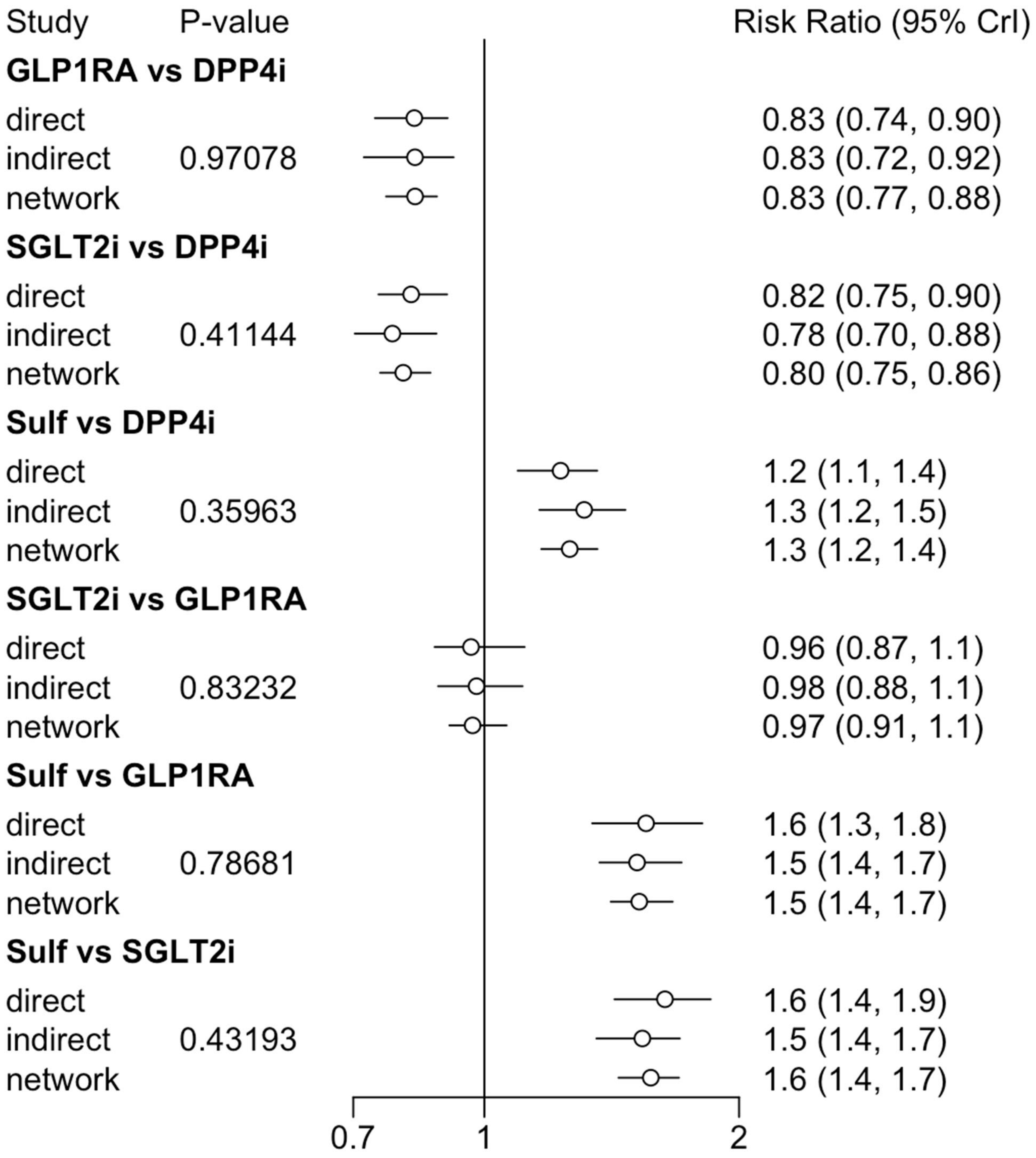

Data are reported as risk ratios and 95% credible interval. Abbreviations: DPP4i: dipeptidyl peptidase-4 inhibitor; GLP1RA: glucagon-like peptide-1 receptor agonist; SGLT2i: sodium-glucose cotransporter-2 inhibitor; Sulf: sulfonylurea; 95% CrI: 95% credible interval;

2.2. Summary estimates for the outcome “severe exacerbation of COPD” according to direct, indirect and network evidence of the NMA

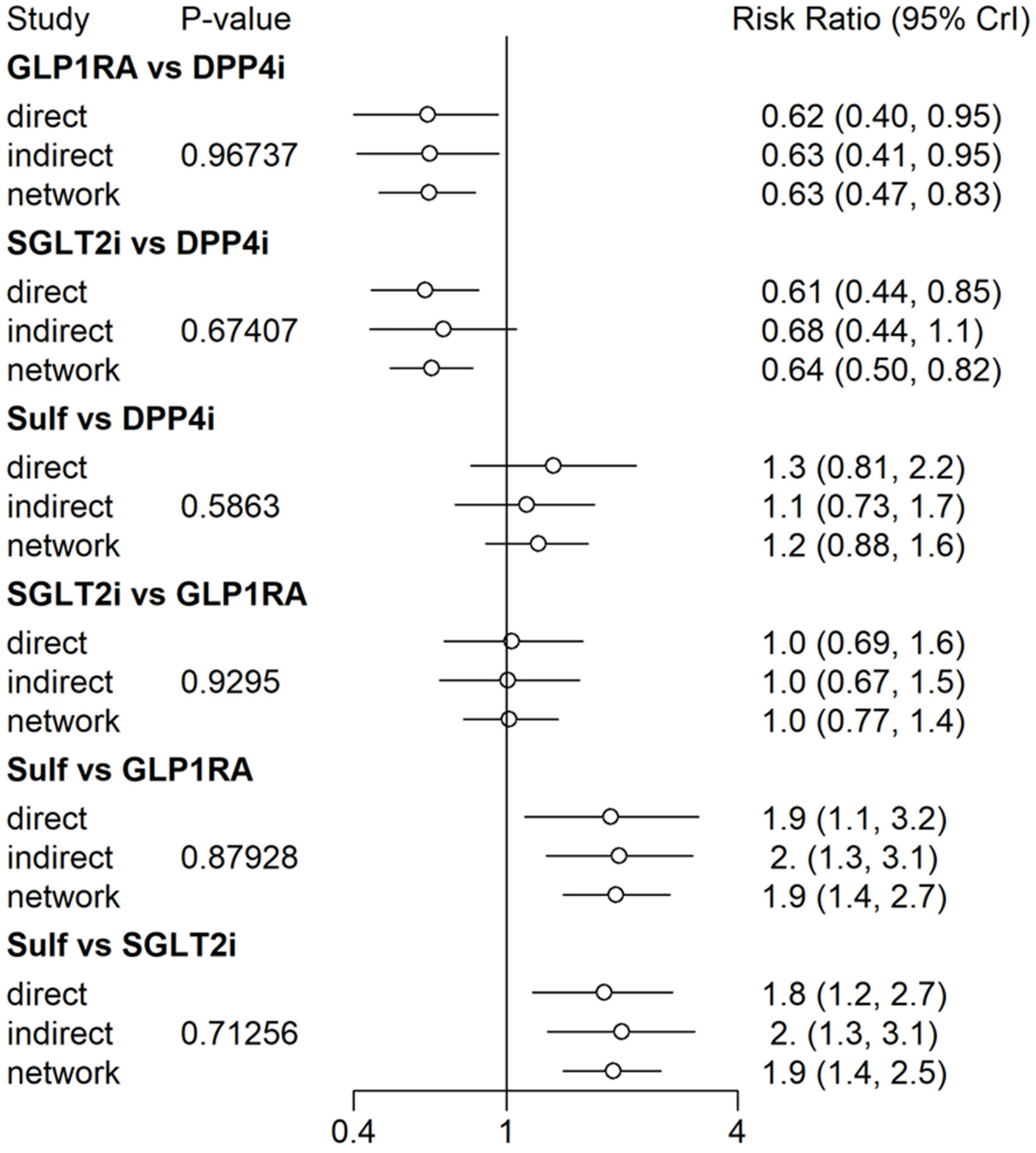

Data are reported as risk ratios and 95% credible interval. Abbreviations: DPP4i: dipeptidyl peptidase-4 inhibitor; GLP1RA: glucagon-like peptide-1 receptor agonist; SGLT2i: sodium-glucose cotransporter-2 inhibitor; Sulf: sulfonylurea; 95% CrI: 95% credible interval;

2.3. Summary estimates for the outcome "moderate exacerbation of COPD" according to direct, indirect and network evidence of the NMA

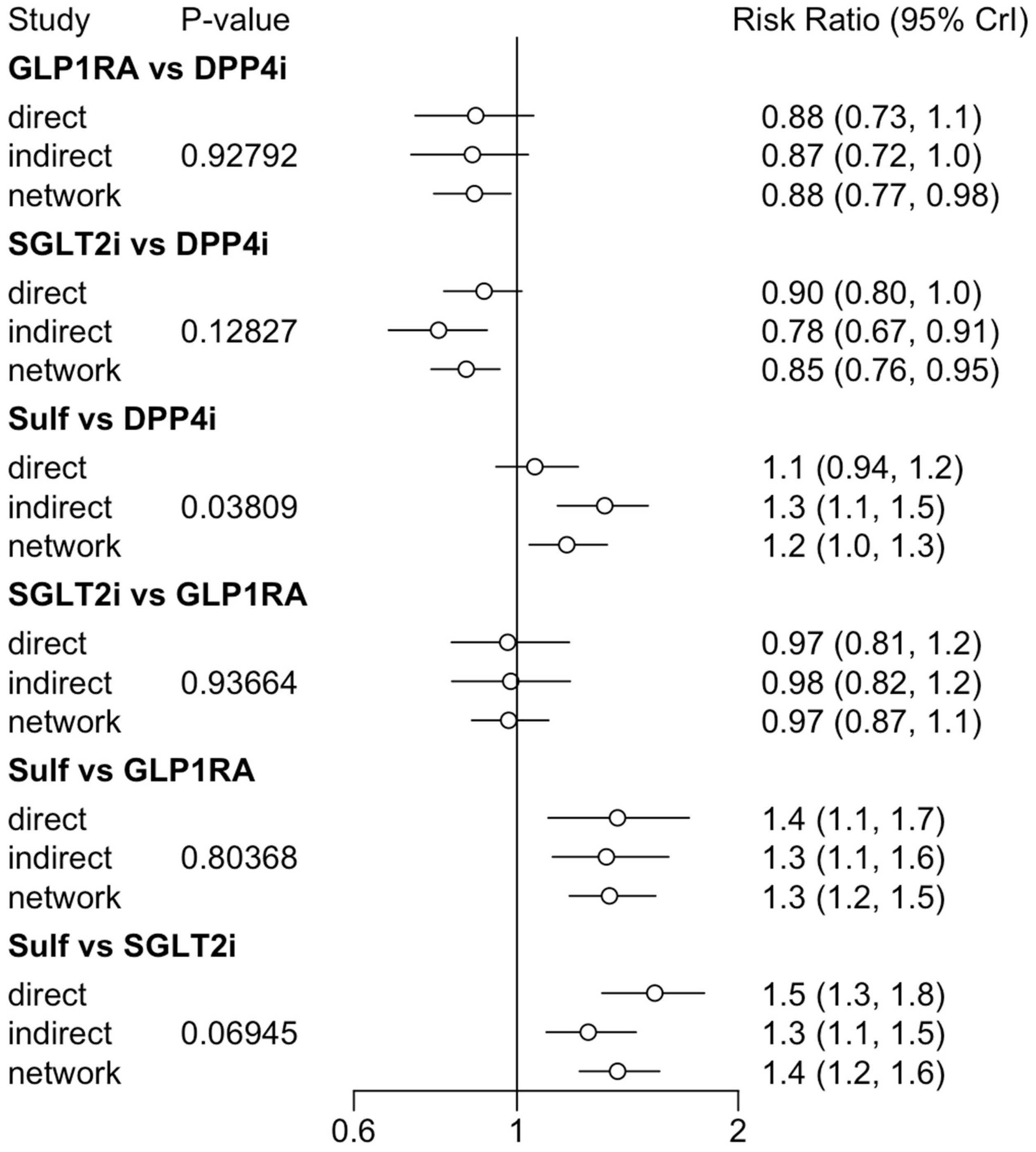

Data are reported as risk ratios and 95% credible interval. Abbreviations: DPP4i: dipeptidyl peptidase-4 inhibitor; GLP1RA: glucagon-like peptide-1 receptor agonist; SGLT2i: sodium-glucose cotransporter-2 inhibitor; Sulf: sulfonylurea; 95% CrI: 95% credible interval;

3. Results of the comparison of Bayesian model fit with and without the assumption of evidence inconsistency and the comparison of Bayesian model fit using different prior distributions

In Bayesian NMA using the gemtc package's default configuration, a non-informative normal prior distribution is assigned to the log-transformed RRs. The package then uses the data to inform the prior distributions' specifications, ensuring they are sufficiently vague while limiting potential bias. This approach has been subject to validation [13].

Since our NMA was predominantly performed with a small number of studies, we evaluated how our treatment effect estimates varied to different prior distributions and without the assumption of evidence inconsistency. The tau parameter was modelled using three different prior distributions: uniform(0, 2), uniform(0, 5), and inverse-gamma(0.01, 0.01) [14]. We compared model performances using the Deviance Information Criterion (DIC) and the estimated between-study effect heterogeneity and its 95% credible interval. DIC values remained consistent across prior specifications except for the model without the assumption of evidence inconsistency, while heterogeneity estimates showed minimal variation except for the inverse-gamma prior. All pairwise treatment comparisons showed consistent effect estimates across the different model specifications, confirming the robustness of findings from the main analysis.

	Default prior distribution	Uniform(0,2)	Uniform(0,5)	Inverse-gamma (0.01,0.01)	Without evidence of inconsistency
DIC[a]	50.27	50.14	50.27	50.44	53.2
Tau[b]	0.04 (0.001-0.11)	0.041 (0.001-0.114)	0.042 (0.001-0.117)	0.088 (0.050-0.155)	0.065 (0.003-0.173)

[a] Deviance Information Criterion (DIC) measures goodness of fit of the model.
[b] Data are reported as standard deviation of the log risk ratio scale.

4. Results of NMA using effect sizes reported among the included studies

We also conducted a sensitivity analysis utilizing effect estimates as reported in the eligible studies. This methodological approach has allowed us to include the confounder-adjusted effect estimates reported in the original studies, many of which were time-to-event measures such as hazard ratios or incidence rate ratios. This was particularly important given the heterogeneity in follow-up durations across studies, as time-to-event estimates better account for differences in observation periods and thus reduce potential bias related to unequal follow-up. However, due to heterogeneity in the reported effect measures across studies, which could potentially introduce imprecision, this analysis was performed exclusively as a sensitivity analysis rather than as part of the main analysis.

4.1. Effect estimates used across the included studies for primary and secondary outcomes

Study and year	*Moderate or severe exacerbation*	*Severe exacerbation*	*Moderate exacerbation*
**Albogami, 2021** [3]	N/A	Adjusted Hazard ratio	N/A
**Pradhan, 2022** [4]	Risk ratio obtained from event data reported	Adjusted Hazard ratio	Adjusted Hazard ratio
**Foer, 2023** [5]	Adjusted Incidence rate ratio	Adjusted Hazard ratio	Adjusted Hazard ratio
**Au, 2023** [6]	N/A	Odds ratio	N/A
**See, 2024** [7]	Adjusted Hazard ratio	N/A	N/A
**Yen, 2024** [8]	N/A	Adjusted Hazard ratio	N/A
**Chang, 2025** [9]	Adjusted Hazard ratio	Adjusted Hazard ratio	Adjusted Hazard ratio
**Ray, 2025** [10]	Adjusted Hazard ratio	Adjusted Hazard ratio	Risk ratio obtained from event data reported
**Yen, 2025** [11]	N/A	Adjusted Hazard ratio	N/A

N/A: not applicable.

4.2. Adjusted effect estimates for COPD exacerbations comparing glucose-lowering agents across included studies

Author and year	*Intervention*	*Comparator*	*COPD exacerbation (Effect size and 95%CI)*
**Albogami, 2021** [3]	GLP-1RA	DPP4i	Severe: HR 0,46 (0,24-0,89)
**Pradhan, 2022** [4]	GLP-1RA	Sulfonylurea	Severe: HR 0,70 (0,49-0,99)<br>Moderate: HR 0,63 (0,43-0,94)
	DPP4i	Sulfonylurea	Severe: HR 0,91 (0,82-1,02)<br>Moderate: HR 0,93 (0,82-1,07)
	SGLT2i	Sulfonylurea	Severe: HR 0,62 (0,48-0,81)<br>Moderate: HR 1,02 (0,83-1,27)

**Foer, 2023** [5]	DPP4i	GLP-1RA	Composite: IRR 1,22 (0,90-1,65)<br>Severe: HR 1,85 (1,12-3,05)<br>Moderate: HR 1,52 (1,09-2,14)
	SGLT2i	GLP-1RA	Composite: IRR 0,94 (0,70-1,26)<br>Severe: HR 1,41 (0,87-2,29)<br>Moderate: HR 1,01 (0,73-1,39)
	Sulfonylurea	GLP-1RA	Composite: IRR 1,59 (1,25-2,03)<br>Severe: HR 2,21 (1,42-3,44)<br>Moderate: HR 2,09 (1,56-2,79)
**Au, 2023** [6]	DPP4i	SGLT2i	Severe: IRR 2,21 (1,42-3,44)
**See, 2024** [7]	GLP-1RA	DPP4i	Composite: HR 0,82 (0,71-0,94)
**Yen, 2024** [8]	GLP-1RA	SGLT2i	Severe: HR 0,72 (0,55-0,96)
**Chang, 2025** [9]	SGLT2i	DPP4i	Composite: HR 0,69 (0,52-0,92)<br>Severe: HR 0,35 (0,20-0,61)<br>Moderate: HR 0,76 (0,57-1,03)
**Ray, 2025** [10]	SGLT2i	DPP4i	Composite: HR 0,81 (0,76-0,86)<br>Severe: HR 0,71 (0,65-0,78)
	GLP-1RA	DPP4i	Composite: HR 0,86 (0,81-0,91)<br>Severe: HR 0,82 (0,76-0,89)
	SGLT2i	GLP-1RA	Composite: HR 0,94 (0,89-1,00)<br>Severe: HR 0,93 (0,85-1,01)
**Yen, 2025** [11]	DPP4i	SGLT2i	Severe: HR 0,96 (0,70-1,31)
	Sulfonylurea	SGLT2i	Severe: HR 0,83 (0,56-1,22)

Data are reported as effect estimates and 95% confidence interval; Abbreviations: DPP4i: dipeptidyl peptidase-4 inhibitor; GLP-1RA: glucagon-like peptide-1 receptor agonist; HR: Hazard ratio; IRR: incidence rate ratio; SGLT2i: sodium-glucose cotransporter-2 inhibitor;

4.3. Matching technique and covariates used for statistical adjustments

Study and year	***Matching Technique***	***Covariates for adjustment of effect size measure***
**Albogami, 2021** [3]	Inverse probability of treatment weights analysis	Sex, Age, Geographic region, Health plan type, Year of drug initiation, Season of drug initiation, Visit to family medicine physicians, Visit to endocrinologist, Visit to pulmonologist, Acquired hypothyroidism, Acute myocardial infarction, Glaucoma, Congestive heart failure, Nonalcoholic fatty liver disease, Hypertension, Stroke, Coronary artery disease, Arrhythmia, Dyslipidemia, Sleep apnea, Obesity, Depression, Pneumonia, Influenza, Hypoglycemia, Total number of hospital visits, Total number of total hospitalizations, History of CLRD-related hospitalizations, Total number of total unique prescriptions dispensing, Total number of total concomitant prescriptions, Number of total emergency department visits, Total number of oral corticosteroids dispensing, Total number of CLRD rescue medications dispensing, Total number of CLRD controller medications dispensing, Oxygen supplement use, Double or triple therapy, Total number of metformin use, Total number of sulfonylurea use, Total number of glitazone use, Total number of SGLT-2I use, Eye-exams, Proteinuria, Blood glucose test.

**Pradhan, 2022** [4]	Propensity score analysis	Age (modelled using cubic splines with five interior knots), sex, body mass index, smoking status, and alcohol related disorders, including haemoglobin A1c, duration of diabetes before cohort entry (defined by the date of the first of a haemoglobin A1c≥6.5%, a diagnosis of type 2 diabetes, or prescription for any antihyperglycaemic drug, modelled using cubic splines with five interior knots), microvascular complications (nephropathy, neuropathy, retinopathy), macrovascular complications (myocardial infarction, stroke, peripheral arteriopathy), antihyperglycaemic drugs, duration of COPD (time between the initial diagnosis and cohort entry), respiratory drugs prescribed in the year before cohort entry (including long acting and short acting β agonist drugs, long acting and short acting anti-muscarinic drugs, inhaled and oral corticosteroids, leukotriene antagonists, methylxanthines, respiratory antibiotics), respiratory events in the year before cohort entry (hospital admission for chronic obstructive pulmonary disease (any position), pneumonia, or influenza), per cent predicted forced expiratory volume in one second (FEV1),and FEV1 to forced vital capacity ratio, and dyspnoea (mMRC), along with blood eosinophil count (<2%, 2-4%, >4%) at cohort entry, asthma, interstitial lung diseases, cystic fibrosis, bronchiectasis, pulmonary embolism, pulmonary hypertension) and lung cancer at any time before cohort entry. Cancer other than non-melanoma skin cancer, heart failure, hypertension, arrhythmia, dyslipidaemia, nonalcoholic fatty liver disease, hypothyroidism, gastrooesophageal reflux disease, obesity, sleep apnoea, osteoarthritis, depression) and drugs prescribed in the year before cohort entry (angiotensin converting enzyme inhibitors, angiotensin receptor blockers, β blockers, calcium channel blockers, thiazides, other diuretics, antiarrhythmic agents, antiplatelet agents, statins, proton pump inhibitors, non-steroidal anti-inflammatory drugs, and opioids), uptake of cancer screening (faecal occult blood testing or colonoscopy, mammography, prostate specific antigen testing), influenza and pneumococcal vaccinations. Season (winter (December-February), spring (March-May), summer (June-August), and fall/autumn (September-November)).
**Foer, 2023** [5]	No matching technique	Age, sex, race, index year, Elixhauser Comorbidity Index, total health system encounter, history, season of initiation, current smoking history, concurrent metformin use, COPD medications. Baseline HbA1c and baseline BMI, treated as continuous variables.
**Au, 2023** [6]	Propensity score analysis	Sex, age at index date, season of index date, angiotensin-converting enzyme inhibitors or angiotensin II receptor blockers, antiarrhythmic agents, anticoagulants, beta-blockers, calcium channel blockers, cardiac glycosides, loop diuretics, other diuretics, nitrates, peripheral vasodilators, platelet inhibitors, bronchodilators, inhaled corticosteroids (including corticosteroid/bronchodilator or corticosteroid/long-acting beta-agonist combinations), antibiotics, immunosuppressants, non-steroidal anti-inflammatory agents, antidepressants, antipsychotics, phosphate-binding agents, lipid-regulating agents, proton pump inhibitors, systemic corticosteroids, hormone replacement therapy, metformin, sulfonylureas, thiazolidinediones, glucagon-like peptide-1 receptor agonists, acarbose, insulin, coronary heart disease, myocardial infarction, cerebrovascular diseases, hypertensive diseases, arrhythmias and conduction disorders, arterial disease, chronic kidney disease (eGFR <60 mL/min/1.73 m²), pneumonia, acute bronchitis, bronchiectasis, extrinsic allergic alveolitis, other lung diseases, obesity, hyperlipidemia, thyroid disease, osteoporosis, osteoporotic fractures, all fractures, Paget's disease, rheumatoid arthritis, cancers, chronic pancreatitis, dementia, liver diseases (chronic liver disease, cirrhosis, esophageal varices, hepatic failure), diabetic eye complications, diabetic hyperosmolarity, diabetic neuropathy, peripheral artery diseases, diabetic ketoacidosis, HbA1c, estimated glomerular filtration rate (eGFR), number of glucose-lowering agents used in the prior five years, days since first diabetes diagnosis, number of emergency admissions in the prior year, number of planned admissions in the prior year, level of maintenance therapy

		for obstructive airway disease (none, inhaled corticosteroids only or long-acting bronchodilators only, inhaled corticosteroids plus long-acting bronchodilators), baseline co-medications (metformin, sulfonylureas, thiazolidinediones, glucagon-like peptide-1 receptor agonists, acarbose, insulin), and total number of baseline co-medications.
**See, 2024** [7]	Propensity score analysis	Age, sex, race, tobacco use, BMI, HbA1c, absolute eosinophil count, FEV1, long-term use of steroids, oxygen dependence, pre-existing comorbidities, medication use for cardiovascular, diabetic and pulmonary diseases.
**Yen, 2024** [8]	Propensity score analysis	Age, sex, smoking status, obesity (the composite diagnoses of overweight, obese and severely obese), comorbidities (hypertension, dyslipidaemia, coronary artery disease, stroke, heart failure, chronic kidney disease, liver cirrhosis, pneumonia and atrial fibrillation) diagnosed within 1 year before the index date, medications (ß2-bronchodilator inhalers, anticholinergic inhalers, corticosteroid inhalers, methylxanthines, oral systemic corticosteroids, insulins, antidiabetic drugs and cardiovascular drugs) and duration of T2D, Charlson Comorbidity Index, DCSI.
**Chang, 2025** [9]	No matching technique	Age, sex, comorbidities, DCSI, history of moderate or severe exacerbation, COPD drug used, and OCS.
**Ray, 2025** [10]	Propensity score analysis	Age, gender, severe COPD, moderate COPD exacerbation, GOLD group E (defined as ≥2 moderate COPD exacerbations and/or ≥1 severe COPD exacerbation in the 365 days prior to cohort entry), corticosteroids (oral), pneumonia, pneumonia hospitalization, active asthma (defined as ≥2 outpatient or ≥1 inpatient asthma diagnosis at any position; asthma, influenza, bronchiectasis, pulmonary hypertension, pulmonary embolism, interstitial lung disease or pulmonary fibrosis, lung cancer, obstructive sleep apnea diagnosis or continuous positive airway pressure/bilevel positive airway pressure machine usage, spirometry performed (yes/no), use of any combination of SABA, SAMA, LABA, LAMA, ICS, inhaler monotherapy for bronchial hyperresponsiveness (defined as use of SABA, SAMA, LABA, LAMA, or ICS in the 365 days prior to cohort entry), dual therapy (defined as use of SABA-SAMA, LABA-LAMA, or LABA-ICS in the 365 days prior to cohort entry), triple therapy (defined as LABA-LAMA-ICS in the 365 days prior to cohort entry), respiratory events in the 60-days prior to cohort entry (including moderate or severe COPD exacerbation, respiratory antibiotics, COPD hospitalization, and oxygen equipment use), combined comorbidity score, frailty index score (categorized as <0.15, 0.15-0.25, ≥0.25), BMI (categorized as <25 kg/m2 for under- or normal weight , 25-29.9 kg/m2 for overweight, and ≥30 kg/m2 for obese), diabetic retinopathy, diabetic neuropathy, PVD or PVD surgery, diabetes with non-specific complications, CHF, hypertension, coronary procedures, coronary atherosclerosis ischemic stroke, acute myocardial infarction, arrhythmias, atrial fibrillation, hyperlipidemia, chronic kidney disease (stage 1-2, stage 3-4, unspecified), NAFLD (including nonalcoholic steatohepatitis or NASH), gastroesophageal reflux disease, hypothyroidism, osteoporosis (without fractures), cancer (excluding lung cancer and non-melanoma skin cancer), anxiety, dementia, and COVID-19
**Yen, 2025** [11]	Propensity score analysis	Age, sex, smoking status, obesity, comorbidities diagnosed within 1 year before the index date, medications, Charlson Comorbidity Index, DCSI, duration of diabetes, and the number of moderate or severe COPD exacerbations.

Abbreviations: ACE: Angiotensin-Converting Enzyme; ARB: Angiotensin II Receptor Blocker; BMI: Body Mass Index; CHF: Congestive Heart Failure; CLRD: Chronic Lower Respiratory Disease; COPD: Chronic Obstructive Pulmonary Disease; COVID-19: Coronavirus Disease 2019; DCSI: Diabetes Complications Severity Index; DPP4i: Dipeptidyl Peptidase-4 Inhibitor; eGFR: Estimated Glomerular Filtration Rate; FEV1: Forced Expiratory Volume in One Second; FVC: Forced Vital Capacity; GOLD: Global Initiative for Chronic Obstructive Lung Disease; HbA1c: Hemoglobin A1c; ICS: Inhaled Corticosteroids; LABA: Long-Acting Beta-Agonist; LAMA: Long-Acting Muscarinic Antagonist; mMRC: Modified Medical Research Council; NAFLD: Non-Alcoholic Fatty Liver Disease; NASH: Non-Alcoholic Steatohepatitis; OCS: Oral Corticosteroids; PVD: Peripheral Vascular Disease; SABA: Short-Acting Beta-Agonist; SABD: Short-Acting Bronchodilator; SAMA: Short-Acting Muscarinic Antagonist; SGLT2i: Sodium-Glucose Cotransporter-2 Inhibitor.

4.4. Summary estimates of the NMA using effect sizes reported among the included studies

4.4.1. Summary estimates for the outcome “moderate or severe exacerbation of COPD”

**DPP4i**	**0.84 (0.78, 0.89)**	**0.78 (0.71, 0.83)**	**1.3 (1.2, 1.42)**
**1.19 (1.12, 1.29)**	**GLP1RA**	**0.93 (0.86, 0.99)**	**1.55 (1.43, 1.71)**
**1.28 (1.2, 1.4)**	**1.08 (1.01, 1.17)**	**SGLT2i**	**1.67 (1.54, 1.85)**
**0.77 (0.7, 0.83)**	**0.64 (0.58, 0.7)**	**0.6 (0.54, 0.65)**	**Sulf**

4.4.2. Summary estimates for the outcome “moderate exacerbation of COPD”

**DPP4i**	**0.77 (0.59, 0.98)**	0.82 (0.64, 1.03)	1.2 (0.93, 1.57)
**1.29 (1.02, 1.7)**	**GLP1RA**	1.06 (0.82, 1.37)	**1.55 (1.2, 2.09)**
1.22 (0.97, 1.57)	0.94 (0.73, 1.21)	**SGLT2i**	**1.46 (1.14, 1.95)**
0.84 (0.64, 1.08)	**0.65 (0.48, 0.83)**	**0.69 (0.51, 0.88)**	**Sulf**

4.4.3. Summary estimates for the outcome “severe exacerbation of COPD”

**DPP4i**	**0.70 (0.51, 0.89)**	**0.74 (0.58, 0.95)**	1.10 (0.81, 1.47)
**1.44 (1.12, 1.96)**	**GLP1RA**	1.07 (0.84, 1.43)	**1.58 (1.20, 2.17)**
**1.35 (1.05, 1.73)**	0.94 (0.70, 1.19)	**SGLT2i**	**1.48 (1.11, 1.95)**
0.91 (0.68, 1.23)	**0.63 (0.46, 0.83)**	**0.68 (0.51, 0.90)**	**Sulf**

Data are reported as effect estimate and 95% credible interval. Data in bold are statistically significant. Abbreviations: DPP4i: dipeptidyl peptidase-4 inhibitor; GLP1RA: glucagon-like peptide-1 receptor agonist; SGLT2i: sodium-glucose cotransporter-2 inhibitor; Sulf: sulfonylurea; 95% CrI: 95% credible interval;

5. Summary estimates of the NMA including only studies judged at "low risk" of bias according to ROBINS-I

5.1.1. Summary estimates for the outcome "moderate or severe exacerbation of COPD"

**DPP4i**	**0.83 (0.75, 0.92)**	**0.78 (0.69, 0.87)**	**1.3 (1.17, 1.48)**
**1.2 (1.09, 1.33)**	**GLP1RA**	0.93 (0.83, 1.04)	**1.56 (1.39, 1.79)**
**1.28 (1.16, 1.45)**	1.07 (0.96, 1.21)	**SGLT2i**	**1.67 (1.49, 1.94)**
**0.77 (0.67, 0.86)**	**0.64 (0.56, 0.72)**	**0.6 (0.52, 0.67)**	**Sulf**

5.1.2. Summary estimates for the outcome "moderate exacerbation of COPD"

**DPP4i**	0.87 (0.61, 1.23)	0.82 (0.57, 1.15)	1.19 (0.84, 1.7)
1.15 (0.81, 1.64)	**GLP1RA**	0.94 (0.66, 1.33)	1.36 (0.96, 1.98)
1.22 (0.87, 1.74)	1.06 (0.75, 1.52)	**SGLT2i**	**1.44 (1.03, 2.09)**
0.84 (0.59, 1.19)	0.73 (0.51, 1.05)	**0.69 (0.48, 0.97)**	**Sulf**

5.1.3. Summary estimates for the outcome "severe exacerbation of COPD"

**DPP4i**	0.68 (0.43, 1.04)	**0.65 (0.44, 0.96)**	1.29 (0.77, 2.09)
1.47 (0.96, 2.33)	**GLP1RA**	0.96 (0.63, 1.49)	**1.91 (1.14, 3.18)**
**1.54 (1.05, 2.29)**	1.05 (0.67, 1.58)	**SGLT2i**	**1.99 (1.25, 3.11)**
0.77 (0.48, 1.29)	**0.52 (0.31, 0.88)**	**0.5 (0.32, 0.8)**	**Sulf**

Data are reported as risk ratios and 95% credible interval. Data in bold are statistically significant. Abbreviations: DPP4i: dipeptidyl peptidase-4 inhibitor; GLP1RA: glucagon-like peptide-1 receptor agonist; SGLT2i: sodium-glucose cotransporter-2 inhibitor; Sulf: sulfonylurea; 95% CrI: 95% credible interval;

References

1. Hutton, B.; Salanti, G.; Caldwell, D.M.; Chaimani, A.; Schmid, C.H.; Cameron, C.; Ioannidis, J.P.A.; Straus, S.; Thorlund, K.; Jansen, J.P.; et al. The PRISMA Extension Statement for Reporting of Systematic Reviews Incorporating Network Meta-Analyses of Health Care Interventions: Checklist and Explanations. *Ann Intern Med* **2015**, *162*, 777–784, doi:10.7326/M14-2385.
2. Stroup, D.F.; Berlin, J.A.; Morton, S.C.; Olkin, I.; Williamson, G.D.; Rennie, D.; Moher, D.; Becker, B.J.; Sipe, T.A.; Thacker, S.B. Meta-Analysis of Observational Studies in Epidemiology: A Proposal for Reporting. Meta-Analysis Of Observational Studies in Epidemiology (MOOSE) Group. *JAMA* **2000**, *283*, 2008–2012, doi:10.1001/jama.283.15.2008.
3. Albogami, Y.; Cusi, K.; Daniels, M.J.; Wei, Y.-J.J.; Winterstein, A.G. Glucagon-Like Peptide 1 Receptor Agonists and Chronic Lower Respiratory Disease Exacerbations Among Patients With Type 2 Diabetes. *Diabetes Care* **2021**, *44*, 1344–1352, doi:10.2337/dc20-1794.
4. Pradhan, R.; Lu, S.; Yin, H.; Yu, O.H.Y.; Ernst, P.; Suissa, S.; Azoulay, L. Novel Antihyperglycaemic Drugs and Prevention of Chronic Obstructive Pulmonary Disease Exacerbations among Patients with Type 2 Diabetes: Population Based Cohort Study. *BMJ* **2022**, *379*, e071380, doi:10.1136/bmj-2022-071380.
5. Foer, D.; Strasser, Z.H.; Cui, J.; Cahill, K.N.; Boyce, J.A.; Murphy, S.N.; Karlson, E.W. Association of GLP-1 Receptor Agonists with Chronic Obstructive Pulmonary Disease Exacerbations among Patients with Type 2 Diabetes. *Am J Respir Crit Care Med* **2023**, *208*, 1088–1100, doi:10.1164/rccm.202303-0491OC.
6. Au, P.C.M.; Tan, K.C.B.; Lam, D.C.L.; Cheung, B.M.Y.; Wong, I.C.K.; Kwok, W.C.; Sing, C.-W.; Cheung, C.-L. Association of Sodium-Glucose Cotransporter 2 Inhibitor vs Dipeptidyl Peptidase-4 Inhibitor Use With Risk of Incident Obstructive Airway Disease and Exacerbation Events Among Patients With Type 2 Diabetes in Hong Kong. *JAMA Netw Open* **2023**, *6*, e2251177, doi:10.1001/jamanetworkopen.2022.51177.
7. See, X.Y.; Xanthavanij, N.; Lee, Y.-C.; Ong, T.E.; Wang, T.H.; Ahmed, O.; Chang, Y.-C.; Peng, C.-Y.; Chi, K.-Y.; Chang, Y.; et al. Pulmonary Outcomes of Incretin-Based Therapies in COPD Patients Receiving Single-Inhaler Triple Therapy. *ERJ Open Res* **2025**, *11*, 00803–02024, doi:10.1183/23120541.00803-2024.
8. Yen, F.-S.; Hsu, C.-C.; Wei, J.C.-C.; Tsai, F.-J.; Huang, Y.; Yu, T.-S.; Hwu, C.-M. Glucagon-like Peptide-1 Receptor Agonists May Benefit Cardiopulmonary Outcomes in Patients with COPD. *Thorax* **2024**, *79*, 1017–1023, doi:10.1136/thorax-2023-221040.
9. Chang, T.-C.; Liang, Y.-C.; Lai, C.-C.; Ho, C.-H.; Chen, Y.-C.; Liao, K.-M.; Liang, F.-W. Comparison of SGLT2 and DPP4 Inhibitors on Clinical Outcomes in COPD Patients with Diabetes: A Nationwide Cohort Study. *Diabetes Res Clin Pract* **2025**, *223*, 112122, doi:10.1016/j.diabres.2025.112122.
10. Ray, A.; Paik, J.M.; Wexler, D.J.; Sreedhara, S.K.; Bykov, K.; Feldman, W.B.; Patorno, E. Glucose-Lowering Medications and Risk of Chronic Obstructive Pulmonary Disease Exacerbations in Patients With Type 2 Diabetes. *JAMA Intern Med* **2025**, e247811, doi:10.1001/jamainternmed.2024.7811.
11. Yen, F.-S.; Wei, J.C.-C.; Huang, Y.-H.; Hsu, T.-J.; Wang, S.-T.; Hwu, C.-M.; Hsu, C.-C. SGLT-2 Inhibitors and the Risk of Chronic Obstructive Pulmonary Disease Exacerbations and Mortality in Chronic Obstructive Pulmonary Disease Patients. *Annals ATS* **2025**, *22*, 846–854, doi:10.1513/AnnalsATS.202407-703OC.

12. Sterne, J.A.; Hernán, M.A.; Reeves, B.C.; Savović, J.; Berkman, N.D.; Viswanathan, M.; Henry, D.; Altman, D.G.; Ansari, M.T.; Boutron, I.; et al. ROBINS-I: A Tool for Assessing Risk of Bias in Non-Randomised Studies of Interventions. *BMJ* **2016**, *355*, i4919, doi:10.1136/bmj.i4919.
13. van Valkenhoef, G.; Lu, G.; de Brock, B.; Hillege, H.; Ades, A.E.; Welton, N.J. Automating Network Meta-Analysis. *Res Synth Methods* **2012**, *3*, 285–299, doi:10.1002/jrsm.1054.
14. Turner, R.M.; Davey, J.; Clarke, M.J.; Thompson, S.G.; Higgins, J.P. Predicting the Extent of Heterogeneity in Meta-Analysis, Using Empirical Data from the Cochrane Database of Systematic Reviews. *Int J Epidemiol* **2012**, *41*, 818–827, doi:10.1093/ije/dys041.
